# Intratumoral injection of the seasonal flu shot converts immunologically cold tumors to hot and serves as an immunotherapy for cancer

**DOI:** 10.1073/pnas.1904022116

**Published:** 2019-12-30

**Authors:** Jenna H. Newman, C. Brent Chesson, Nora L. Herzog, Praveen K. Bommareddy, Salvatore M. Aspromonte, Russell Pepe, Ricardo Estupinian, Mones M. Aboelatta, Stuti Buddhadev, Saeed Tarabichi, Michael Lee, Shengguo Li, Daniel J. Medina, Eileena F. Giurini, Kajal H. Gupta, Gabriel Guevara-Aleman, Marco Rossi, Christina Nowicki, Abdulkareem Abed, Josef W. Goldufsky, Joseph R. Broucek, Raquel E. Redondo, David Rotter, Sachin R. Jhawar, Shang-Jui Wang, Frederick J. Kohlhapp, Howard L. Kaufman, Paul G. Thomas, Vineet Gupta, Timothy M. Kuzel, Jochen Reiser, Joyce Paras, Michael P. Kane, Eric A. Singer, Jyoti Malhotra, Lisa K. Denzin, Derek B. Sant’Angelo, Arnold B. Rabson, Leonard Y. Lee, Ahmed Lasfar, John Langenfeld, Jason M. Schenkel, Mary Jo Fidler, Emily S. Ruiz, Amanda L. Marzo, Jai S. Rudra, Ann W. Silk, Andrew Zloza

**Affiliations:** ^a^Rutgers Cancer Institute of New Jersey, Rutgers, The State University of New Jersey, New Brunswick, NJ 08901;; ^b^Department of Surgery, Rutgers Robert Wood Johnson Medical School, Rutgers, The State University of New Jersey, New Brunswick, NJ 08901;; ^c^Department of Internal Medicine, Section of Hematology, Oncology, and Cell Therapy, Rush University Medical Center, Chicago, IL 60612;; ^d^Institute for Genomic Medicine, Columbia University Irving Medical Center, New York, NY 10032;; ^e^Department of Surgery, Vanderbilt University Medical Center, Nashville, TN 37232;; ^f^Department of Surgery, Franciscan Health, Munster, IN 46321;; ^g^Department of Radiation Oncology, Arthur G. James Comprehensive Cancer Center, The Ohio State University, Columbus, OH 43210;; ^h^Division of Surgical Oncology, Massachusetts General Hospital, Boston, MA 02114;; ^i^Replimune, Inc., Woburn, MA 01801;; ^j^Department of Immunology, St. Jude Children’s Research Hospital, Memphis, TN 38105;; ^k^Integrated Biomedical Sciences Program, University of Tennessee Health Science Center, Memphis, TN 38105;; ^l^Child Health Institute of New Jersey, Rutgers Robert Wood Johnson Medical School, New Brunswick, NJ 08901;; ^m^Department of Pharmacology and Toxicology, Ernest Mario School of Pharmacy, Rutgers, The State University of New Jersey, Piscataway, NJ 08854;; ^n^Koch Institute for Integrative Cancer Research, Massachusetts Institute of Technology, Cambridge, MA 02139;; ^o^Department of Pathology, Brigham and Women’s Hospital, Boston, MA 02115;; ^p^Department of Dermatology, Brigham and Women’s Hospital, Boston, MA 02130;; ^q^Harvard Medical School, Boston, MA 02115;; ^r^Department of Biomedical Engineering, McKelvey School of Engineering, Washington University in Saint Louis, Saint Louis, MO 63105;; ^s^Department of Medical Oncology, Dana–Farber Cancer Institute, Boston, MA 02215

**Keywords:** influenza, vaccine, cancer, intratumoral, flu shot

## Abstract

Reprogramming the tumor microenvironment to increase immune-mediated responses is currently of intense interest. Patients with immune-infiltrated “hot” tumors demonstrate higher treatment response rates and improved survival. However, only the minority of tumors are hot, and a limited proportion of patients benefit from immunotherapies. Innovative approaches that make tumors hot can have immediate impact particularly if they repurpose drugs with additional cancer-unrelated benefits. The seasonal influenza vaccine is recommended for all persons over 6 mo without prohibitive contraindications, including most cancer patients. Here, we report that unadjuvanted seasonal influenza vaccination via intratumoral, but not intramuscular, injection converts “cold” tumors to hot, generates systemic CD8^+^ T cell-mediated antitumor immunity, and sensitizes resistant tumors to checkpoint blockade. Importantly, intratumoral vaccination also provides protection against subsequent active influenza virus lung infection. Surprisingly, a squalene-based adjuvanted vaccine maintains intratumoral regulatory B cells and fails to improve antitumor responses, even while protecting against active influenza virus lung infection. Adjuvant removal, B cell depletion, or IL-10 blockade recovers its antitumor effectiveness. Our findings propose that antipathogen vaccines may be utilized for both infection prevention and repurposing as a cancer immunotherapy.

## Significance

Immunotherapy has revolutionized cancer treatment, yielding unprecedented long-term responses and survival. However, a significant proportion of patients remain refractory, which correlates with the absence of immune-infiltrated (“hot”) tumors. Here, we observed that FDA-approved unadjuvanted seasonal influenza vaccines administered via intratumoral injection not only provide protection against active influenza virus lung infection, but also reduce tumor growth by increasing antitumor CD8^+^ T cells and decreasing regulatory B cells within the tumor. Ultimately, intratumoral unadjuvanted seasonal influenza vaccine converts immunologically inactive “cold” tumors to “hot,” generates systemic responses, and sensitizes resistant tumors to checkpoint blockade. Repurposing the “flu shot” may increase response rates to immunotherapy, and based on its current FDA approval and safety profile, may be quickly translated for clinical care.

The tumor microenvironment represents a significant barrier that restricts immune responses against tumors and limits the efficacy of currently available immunotherapies as treatments for cancer. However, immune infiltration of tumors, especially by CD8^+^ T cells, has been shown to correlate with augmented responses to immunotherapy and improved survival (1–5). An immunologically inflamed (“hot”) tumor microenvironment exhibits robust antigen presentation and T cell activation, contributing to the development of tumor-specific CD8^+^ T cell functionality that can acutely eliminate cancer cells, generate systemic tumor-specific immunity, and form long-term antitumor memory responses (5, 6). However, a significant proportion of patients harbor an immunologically “cold” tumor microenvironment that is either devoid of immune cell infiltration (an “immune desert”) or that is predominantly infiltrated by suppressive regulatory cell subtypes (including regulatory T cells [Tregs], regulatory B cells [Bregs], and myeloid-derived suppressor cells [MDSCs]) (7–9). In both environments, cancer growth is immunologically unchecked and recruitment of inflammatory immune cells into such tumors is imperative for antitumor responses. Recently, cancer immunotherapy, including blockade of inhibitory immune checkpoints (such as PD-1/PD-L1 and CTLA-4), has emerged as an unprecedented breakthrough for the treatment of cancer that can induce long-term tumor regression (10–12). However, responses to such therapies have been demonstrated to be effective only in select patients, particularly those who harbor a hot tumor microenvironment (13). Therefore, to increase response rates to immunotherapy, innovative solutions are needed to convert cold tumor microenvironments to hot by increasing infiltration of inflammatory immune cells that can serve as targets for immunotherapies in tumors devoid of immune infiltration and can overcome local immunosuppression in tumors infiltrated by regulatory cells.

One approach that could be utilized involves inducing a strong immune response, unrelated to the immune response against the cancer, within the tumor microenvironment that could then serve as a catalyst for a strong tumor-specific immune response. This concept employs a basic tenet of immunology, that responses against foreign antigens are strong and that responses against self-antigens are inherently weak. Toward avoiding autoimmunity, the immune system has developed many tolerance mechanisms by which strong responses to self-antigens are prevented or eliminated (14–16). Because tumors develop from initially normal cells, many of the antigens of the tumor are self-antigens or antigens similar to self-antigens, and mounting an effective immune response against such antigens is a challenge. This undertaking is made even more difficult by the immunosuppressive nature of the tumor, which increases the immune-activation threshold necessary to be reached before tolerance is broken and potent responses to tumor antigens are mounted. However, when recognizing foreign components (like those associated with pathogens), the immune system is capable of developing strong responses even within the tumor microenvironment (17), and thus, components of pathogens (which can engage receptors associated with innate immunity) may be able to help break tolerance to tumor antigens and improve cancer outcomes.

Here, we show that indeed, pathogens and their components can augment an antitumor immune response within the tumor microenvironment, ultimately converting immunologically cold tumors to hot. This results in inflammatory responses at the injection site that reduce local tumor growth, in augmented systemic antitumor immunity that decreases metastases, and in sensitization of resistant tumors to immune checkpoint blockade. Importantly, we demonstrate that such outcomes can be achieved by intratumoral (i.t.), but not intramuscular, injection of FDA-approved unadjuvanted seasonal influenza vaccines (i.e., “flu shots”), and we elucidate immune mechanisms underlying our observations in the context of multiple mouse and human cancers.

## Results

### Active Influenza Virus Infection in the Lung Improves Outcomes in Mice and Patients with Tumors in the Lung.

**Fig. 1.**
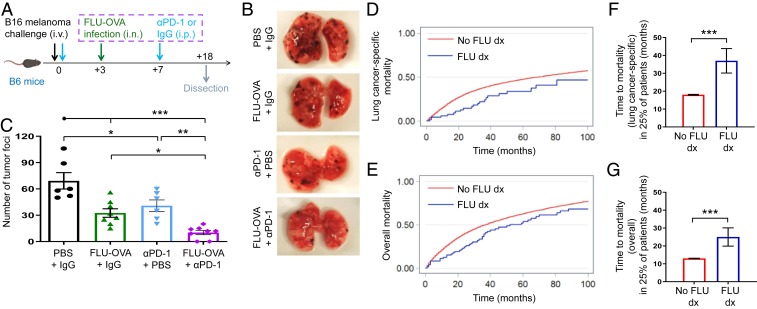
Active influenza virus infection in the lung improves outcomes in mice and patients with tumors in the lung. (*A*) Experimental design. *n* = 6 to 8 lung surfaces/group. Data are representative of at least 2 independent experiments with similar results. (*B*) Representative lung images showing melanoma foci from experiment described in *A*. (*C*) Bar graph showing number of melanoma foci per lung surface from experiment described in *A*. (*D*) Curves of lung cancer-specific mortality in patients with lung cancer included in the SEER-Medicare linked database and followed for 100 mo, who had a recorded hospitalization for influenza virus infection (FLU dx) or not (No FLU dx) during the course of their lung cancer. *n* = 34,277 patients. (*E*) As in *D*, but assessing overall mortality. *n* = 34,529 patients. (*F*) Bar graphs showing mean time to lung cancer-specific mortality in 25% of patients (P25) from database described in *D*. (*G*) As in *F*, but assessing overall mortality. *P < 0.05, **P < 0.01, ***P < 0.001 [1-way ANOVA with Tukey correction (*C*), 2-tailed Student *t* test (*F* and *G*)]. Error bars: mean ± SEM. i.v., intravenous; i.n., intranasal; i.p., intraperitoneal; FLU-OVA, active influenza virus expressing SIINFEKL peptide from ovalbumin (OVA_257–264_); FLU, active influenza virus; IgG, control isotype antibody; αPD-1, PD-1 blocking antibody.

Reports describing cancer outcomes in the context of infection have been discordant (18–21). We recently reported that active influenza virus infection in the lung accelerates early melanoma growth in the skin (22). Further, we showed that antitumor CD8^+^ T cells are shunted from the tumor site (skin) to the distant infection site (lung), resulting in decreased immunity within the tumor, thus permitting accelerated tumor growth (22). Based on these findings and inspired by the previous work of others demonstrating improved anticancer outcomes by targeting pathogens to tumors (23–27), we hypothesized that infection in the same tissue as the tumor (even if the pathogen were not infecting tumor cells directly) would shunt immune cells to this “shared” site of infection and tumor, and thus inflame the tumor microenvironment to reduce tumor growth and prolong host survival. To test our hypothesis, we utilized the same tumor and infection models as our previous report (22), but here, challenged C57BL/6J (B6) mice with B16-F10 melanoma via i.v. injection to localize the tumor to the lung. Concurrently, we administered intranasal (i.n.) injection of FLU-OVA (active influenza A/PR8/1934/H1N1 virus expressing OVA_257–264_ peptide, SIINFEKL) to create a productive infection in the lung (i.e., the same tissue as the tumor). Indeed, active influenza virus infection in the lung reduced melanoma foci in the lung, and this effect was augmented in combination with PD-1 checkpoint blockade (Fig. 1 *A*–*C*). To determine whether our findings have corresponding clinical relevance, we surveyed the Surveillance, Epidemiology, and End Results (SEER)-Medicare linked database of over 30,000 patients with lung cancer. We found that patients who had 1 or more hospitalizations for influenza virus infection during their lung cancer course exhibited decreased lung cancer-specific and overall mortality (Fig. 1 *D* and *E*), in agreement with our mouse model observations. Importantly, the time to lung cancer-specific mortality and overall mortality in 25% of each population was prolonged 12 and 19 mo, respectively, for patients with 1 or more hospitalizations for influenza virus infection during their lung cancer course (Fig. 1 *F* and *G*).

### Intratumoral Heat-Inactivated, but Not Active, Influenza Virus Administration Reduces Tumor Growth in the Skin.

**Fig. 2.**
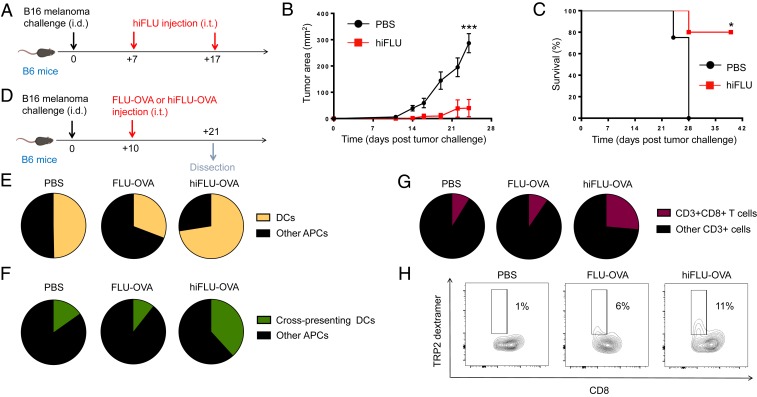
Intratumoral heat-inactivated influenza virus administration reduces tumor growth in the skin and increases cross-presenting DCs and tumor antigen-specific CD8^+^ T cells in the tumor. (*A*) Experimental design. *n* = 4 to 5 mice/group. Data are representative of at least 2 independent experiments with similar results. (*B*) Tumor growth curves from experiment described in *A*. (*C*) Survival curves from experiment described in *A*. (*D*) Experimental design. *n* = 3 to 5 tumors pooled/group. Data are representative of at least 2 independent experiments with similar results. (*E*) Cumulative pie charts of DCs (CD11c^+^) among intratumoral APCs (CD45^+^MHC-II^+^) from experiment described in *D*. (*F*) Cumulative pie charts of cross-presenting dendritic cells (CD11c^+^CD8a^+^) among intratumoral APCs (CD45^+^MHC-II^+^) from experiment described in *D*. (*G*) Cumulative pie charts of CD8^+^ T cells (CD8^+^) among intratumoral T cells (CD45^+^CD3^+^) from experiment described in *D*. (*H*) Cumulative flow cytometry plots of tumor antigen-specific CD8^+^ T cells (TRP2-dextramer^+^) among intratumoral CD8^+^ T cells (CD45^+^CD3^+^CD8^+^) from a similar experiment as described in *D*. *P < 0.05, ***P < 0.001 [2-way ANOVA with Bonferroni correction (*B*), Mantel–Cox log rank test (*C*)]. Error bars: mean ± SEM. i.d., intradermal; i.t., intratumoral; FLU-OVA, active influenza virus expressing SIINFEKL peptide from ovalbumin (OVA_257–264_); hiFLU-OVA, heat-inactivated influenza virus (hiFLU) expressing SIINFEKL peptide from ovalbumin (OVA_257–264_).

Since we determined that active influenza virus administration in the lung reduces melanoma tumors in the lung, and since melanomas form most frequently in the skin, we sought to translate our findings to this more prevalent site. However, intratumoral injection of active influenza virus did not alter skin melanoma growth or host survival (*SI Appendix*, Fig. S1). We hypothesized that since the skin lacks the natural targets for active influenza virus infection that are present in the lung, such virus injected into the skin may be cleared without productive infection of cells within the skin. In this situation, pathways of immune activation including toll-like receptor (TLR)-mediate pathways that are otherwise initiated by the recognition of pathogen-associated molecular patterns (PAMPs) may not be engaged. Further, previous work has shown that active influenza virus injection in the skin dysregulates dendritic cells (DCs) (28). To bypass these scenarios, we created inactivated versions of our active influenza via heat inactivation and chemical lysis. Indeed, when compared to active influenza virus, a heat-inactivated version of our A/PR8/1934/H1N1 active influenza virus (hereafter referred to as hiFLU), demonstrated augmented TLR7 activity, which is produced in response to single-stranded RNA (ssRNA), a natural agonist that constitutes influenza virus (*SI Appendix*, Fig. S2). Importantly, intratumoral administration of hiFLU (or influenza lysate [FLU lysate] likewise derived from active influenza A/PR8/1934/H1N1 virus) reduced tumor growth and prolonged host survival (Fig. 2 *A*–*C* and *SI Appendix*, Fig. S3). Intratumoral hiFLU administration also increased DCs among antigen presenting cells (APCs) in the tumor and, specifically, cross-presenting CD8^+^ DCs (Fig. 2 *D*–*F* and *SI Appendix*, Fig. S4) that have been shown to be important in antitumor and antipathogen (including oncolytic virus) immune responses (29, 30). Further, heat-inactivated influenza virus increased antigen presentation by DCs within the tumor, as demonstrated utilizing hiFLU-OVA, and identifying (via an H-2Kb-OVA antibody) cells presenting SIINFEKL within B6 mouse major histocompatibility complex (MHC) class I molecule, H-2Kb (*SI Appendix*, Fig. S5 *A* and *B*). In *Batf3*^*−/−*^ mice, which lack cross-presenting DCs (29), intratumoral hiFLU-OVA had no effect on tumor growth, thus demonstrating their necessity (*SI Appendix*, Fig. S5 *C* and *D*). Consistent with these findings, we observed increased intratumoral CD8^+^ T cells, and importantly, antitumor CD8^+^ T cells within the tumor microenvironment after hiFLU or hiFLU-OVA administration (Fig. 2 *G* and *H* and *SI Appendix*, Figs. S6 and S7). These findings demonstrate that an inactivated influenza virus can be utilized in the context of the tumor microenvironment to augment an antitumor immune response.

### Heat-Inactivated Influenza Virus Promotes Systemic Antitumor Immunity, Reduces Tumor Growth in Hosts Previously Infected with Active Lung Influenza Virus, and Augments Checkpoint Blockade Immunotherapy.

To determine whether the sum of the mechanistic changes we observed with intratumoral heat-inactivated injection provides systemic immunity, we conducted a bilateral flank experiment. Indeed, both the hiFLU-treated (injected) right and untreated (noninjected) left flank tumors exhibited reduced melanoma growth (*SI Appendix*, Fig. S8 *A*–*C*), suggesting that local intratumoral hiFLU augments systemic antitumor responses. A similar systemic outcome was observed in the 4T1 model of metastatic triple-negative breast cancer, where both primary tumor growth and lung metastases were reduced after intratumoral injection of hiFLU only into the primary tumor (*SI Appendix*, Fig. S8 *D*–*F*), suggesting that intratumoral hiFLU positive antitumor outcomes are not limited to skin cancers or to nonmetastatic tumors. Importantly, intratumoral hiFLU similarly decreased melanoma growth in hosts previously infected with influenza virus (*SI Appendix*, Fig. S8 *G*–*I*), suggesting that intratumoral hiFLU as a treatment for cancer may be utilized in hosts that have been previously infected by and have cleared the same pathogen. To determine whether intratumoral hiFLU could augment checkpoint blockade immunotherapy, hiFLU and PD-L1 blockade were administered in combination. Combination treatment with PD-L1 checkpoint blockade in the melanoma model further reduced tumor growth, compared to that observed with either hiFLU or PD-L1 blockade alone (*SI Appendix*, Fig. S8 *J* and *K*). This suggests that patients who respond (even partially) to such checkpoint blockade may benefit further from administration of intratumoral heat-inactivated influenza virus.

### Unadjuvanted Seasonal Influenza Vaccine Administered via Intratumoral Injection Reduces Growth of Mouse and Human Cancers and Makes Resistant Tumors Responsive to Checkpoint Blockade Immunotherapy.

**Fig. 3.**
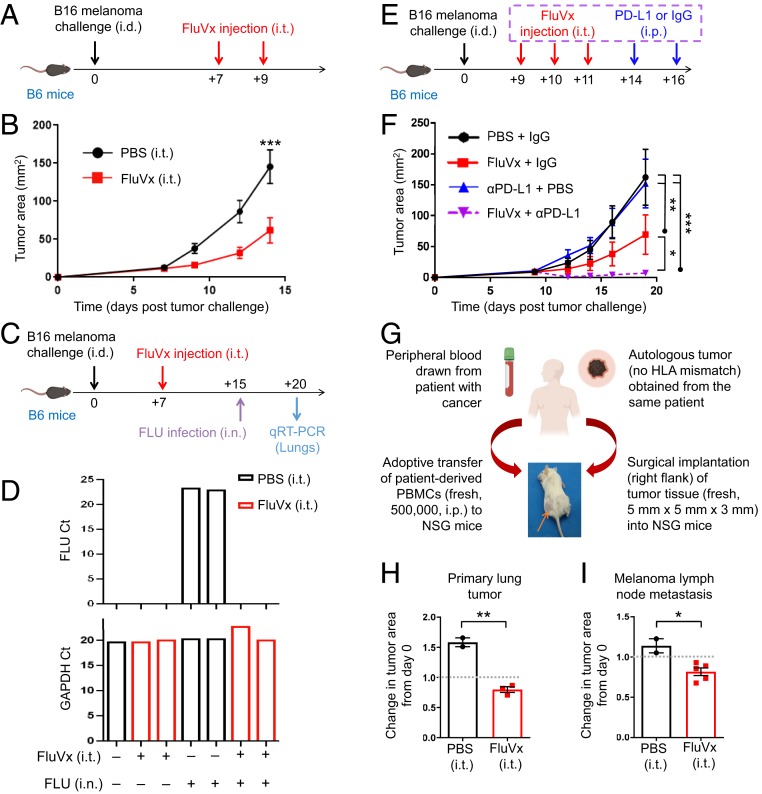
Intratumoral unadjuvanted seasonal influenza vaccine administration reduces tumor growth, augments checkpoint blockade immunotherapy, and protects against active influenza virus lung infection. (*A*) Experimental design. Unadjuvanted seasonal influenza vaccine (FluVx): FluVx1. *n* = 9 mice/group. Data are representative of at least 2 independent experiments with similar results. (*B*) Tumor growth curves from experiment described in *A*. (*C*) Experimental design. FluVx: FluVx1. (*D*) Bar graphs showing count threshold (Ct) of active influenza virus (FLU) or GAPDH control qRT-PCR transcripts from experiment described in *C*. (*E*) Experimental design. FluVx: FluVx1. *n* = 4 to 5 mice/group. Data are representative of at least 2 independent experiments with similar results. (*F*) Tumor growth curves from experiment described in *E*. (*G*) Schematic describing development of the AIR-PDX model in which NSG mice receive adoptive transfer of fresh patient-derived human peripheral blood mononuclear cells (PBMCs; 500,000 cells) and surgically implanted tumor sections (∼5 mm × 5 mm × 3 mm) from the same (i.e., autologous) patient. FluVx: FluVx1. *n* = 2 to 5 mice/group. (*H*) Bar graphs showing change in area of a primary lung tumor (day 13 area/day 0 area) from experiment described in *G*. Dotted line corresponds to day 0 (first treatment day). (*I*) Bar graphs showing change in area of a melanoma lymph node metastasis (day 16 area/day 0 area) from experiment described in *G*. Dotted line corresponds to day 0 (first treatment day). *P < 0.05, **P < 0.01, ***P < 0.001 [2-way ANOVA with Bonferroni correction (*B*) or Tukey correction (*F*), 2-tailed Student *t* test (*H* and *I*)]. Error bars: mean ± SEM. i.d., intradermal; i.t., intratumoral; i.p., intraperitoneal; i.n., intranasal. IgG, control isotype antibody; αPD-L1, PD-L1 blocking antibody.

Based on the successes of the inactivated influenza viruses (that we produced from active influenza virus) in reducing tumor growth, and with clinical translatability in mind, we hypothesized that commercially available seasonal influenza vaccines (flu shots) could be repurposed for cancer immunotherapy, as the majority of these vaccines are inactivated like the hiFLU we produced. Indeed intratumoral, but not intramuscular, injection of the 2017–2018 unadjuvanted seasonal influenza vaccine (FluVx, *SI Appendix*, Table S1) resulted in reduced tumor growth (Fig. 3 *A* and *B* and *SI Appendix*, Figs. S9 and S10 *A* and *B*). Multiple FluVx administrations further reduced tumor growth (*SI Appendix*, Fig. S10 *C* and *D*), suggesting a role here for the prime-boost approach used for children receiving their initial influenza vaccination. Importantly, intratumoral injection of FluVx afforded hosts protection against subsequent active influenza virus infection (Fig. 3 *C* and *D*), suggesting that administration of an unadjuvanted seasonal influenza vaccine in the tumor may be used to simultaneously reduce tumor growth and provide vaccination-induced protection against influenza virus lung infection. The combination of FluVx with PD-L1 checkpoint blockade further reduced tumor growth, even in the context where the tumor was resistant to checkpoint blockade alone (Fig. 3 *E* and *F*). To gauge the possible effect of intratumoral FluVx administration on patient tumors, we utilized the autologous immune-reconstituted patient-derived xenograft (AIR-PDX) mouse model that we have developed. AIR-PDX mice harbor surgically transplanted patient tumor tissue (thus, maintaining the natural architecture of the patient’s tumor) and adoptively transplanted peripheral blood immune cells from the same (autologous) patient (Fig. 3*G*) (thus not requiring stem cells and avoiding mismatched immunity in the tumor versus peripheral blood and tissues). In this model, intratumoral FluVx likewise reduced growth of a patient-derived primary lung tumor and patient-derived melanoma lymph node metastasis (Fig. 3 *H* and *I*), suggesting the translatability of our findings to clinical cancer treatment.

### Intratumoral Unadjuvanted Seasonal Influenza Vaccine Increases the Proportion of Intratumoral Dendritic Cells and Tumor Antigen-Specific CD8^+^ T Cells within the Tumor Microenvironment.

**Fig. 4.**
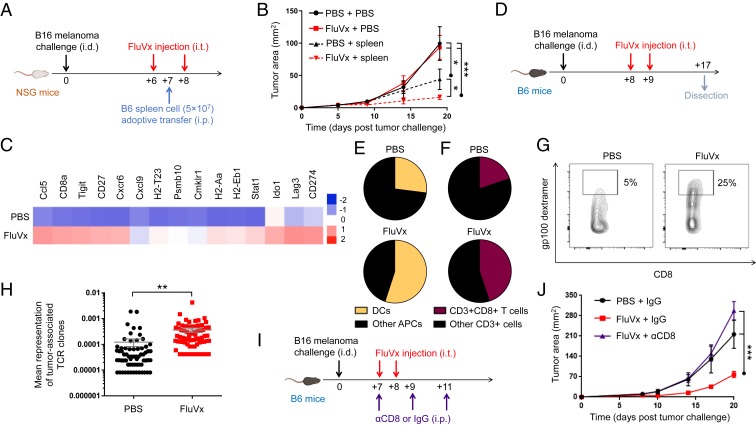
Intratumoral unadjuvanted seasonal influenza vaccine administration produces an immunologically hot tumor microenvironment and increases DCs and tumor antigen-specific CD8^+^ T cells in the tumor. (*A*) Experimental design. Unadjuvanted seasonal influenza vaccine (FluVx): FluVx1. *n* = 3 to 5 mice/group. Data are representative of at least 2 independent experiments with similar results. (*B*) Tumor growth curves from experiment described in *A*. (*C*) Representative heatmap of a focused NanoString PanCancer immune profiling analysis of tumors 7 d posttreatment. FluVx: FluVx1. (*D*) Experimental design. FluVx: FluVx2. *n* = 3 to 5 tumors pooled/group. Data are representative of at least 2 independent experiments with similar results. (*E*) Cumulative pie charts of DCs (CD11c^+^) among intratumoral APCs (CD45^+^ MHC-II^+^) from experiment described in *D*. (*F*) Cumulative pie charts of CD8^+^ T cells (CD8^+^) among intratumoral T cells (CD45^+^CD3^+^) from experiment described in *D*. (*G*) Cumulative flow cytometry plots of tumor antigen-specific (gp100 dextramer^+^) CD8^+^ T cells among intratumoral CD8^+^ T cells (CD45^+^CD3^+^CD8^+^) from a similar experiment as described in *D*. (*H*) Scatterplot from TCR sequencing. FluVx: FluVx1. (*I*) Experimental design. FluVx: FluVx1. *n* = 3 to 4 mice/group. (*J*) Tumor growth curves from experiment described in *I*. *P < 0.05, **P < 0.01, ***P < 0.001 [2-way ANOVA with Tukey correction (*B* and *J*), 2-tailed Student *t* test (*H*)]. Error bars: mean ± SEM. i.d., intradermal; i.t., intratumoral; i.p., intraperitoneal; IgG, control isotype antibody; αCD8, CD8 depleting antibody.

Toward defining mechanisms underlying our FluVx findings, we determined the contribution of the immune system to our observed outcomes. In nonobese diabetic (NOD) severe combined immunodeficiency (*scid*) IL-2 receptor common gamma chain null (gamma) mice (NSG mice), which lack a functional immune system (31), FluVx had no effect on tumor growth; however, immune reconstitution of NSG mice via adoptive cell transfer of splenic-derived immune cells fully recovered the antitumor effect of FluVx (Fig. 4 *A* and *B*), suggesting that the immune system is required for FluVx’s ability to reduce tumor growth. A focused analysis of inflammation-related mRNAs previously shown to correlate with clinical response in patients to PD-1 checkpoint blockade (5) demonstrated high expression of such mRNAs with intratumoral FluVx administration (Fig. 4*C* and Dataset S1), suggesting conversion of an immunologically cold tumor to hot. As with hiFLU, we observed with FluVx an increase in DCs among all APCs in the tumor and a corresponding increase in intratumoral CD8^+^ T cells (Fig. 4 *D*–*F* and *SI Appendix*, Fig. S11). Importantly, among CD8^+^ T cells, we observed an increase in tumor antigen-specific CD8^+^ T cells (Fig. 4*G*), suggesting that intratumoral antipathogen vaccination boosts tumor-specific immunity. Consistent with these findings, and further suggesting that FluVx augments antitumor T cell responses, T cell receptor (TCR) sequencing demonstrated an increase in the representation of tumor-associated clones (i.e., increased evenness/clonality) with intratumoral FluVx (Fig. 4*H* and Dataset S2), a therapy-induced change previously reported in patients responding to PD-1 checkpoint blockade (32). Importantly, depletion of CD8-expressing cells completely abrogated the FluVx antitumor effect (Fig. 4 *I* and *J*), demonstrating the importance of such cells in the underlying immune mechanism of FluVx.

### A Seasonal Influenza Vaccine that Is Adjuvanted Fails to Reduce Tumor Growth due to Maintenance of Regulatory B Cells.

**Fig. 5.**
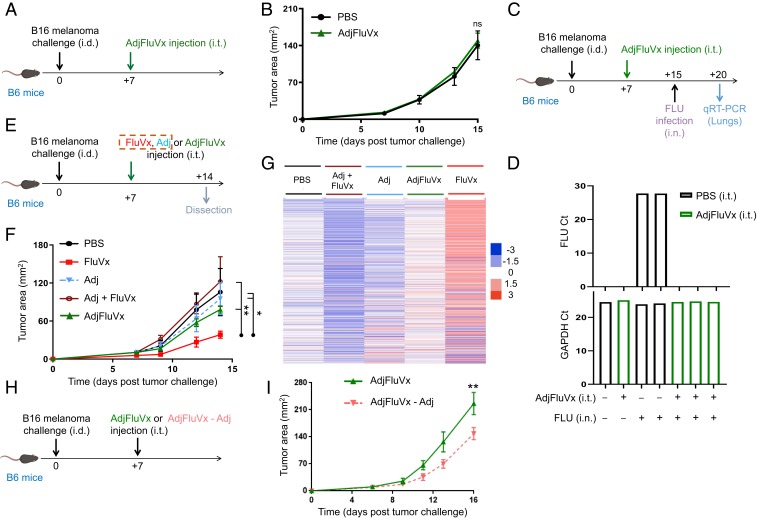
Intratumoral adjuvanted seasonal influenza vaccine administration does not reduce tumor growth but does protect against active influenza virus infection and reduces tumor growth upon removal of its adjuvant. (*A*) Experimental design. *n* = 9 to 10 mice/group. Data are representative of at least 2 independent experiments with similar results. (*B*) Tumor growth curves from experiment described in *A*. (*C*) Experimental design. (*D*) Bar graphs showing count threshold (Ct) of active influenza virus (FLU) or GAPDH control qRT-PCR transcripts from experiment described in *C*. (*E*) Experimental design. Unadjuvanted seasonal influenza vaccine (FluVx): FluVx1. *n* = 3 to 4 mice/group. (*F*) Tumor growth curves from experiment described in *E*. (*G*) Representative heatmap of NanoString PanCancer immune profiling analysis of tumors 7 d posttreatment from experiment described in *E*. (*H*) Experimental design. *n* = 3 mice/group. (*I*) Tumor growth curves from experiment described in *H*. ns, not significant, *P < 0.05, **P < 0.01 [2-way ANOVA with Bonferroni correction (*B* and *I*) or Tukey correction (*F*)]. Error bars: mean ± SEM. i.d., intradermal; i.t., intratumoral; i.n., intranasal; AdjFluVx, adjuvanted seasonal influenza vaccine; Adj, adjuvant; Adj + FluVx, Adj added to FluVx; AdjFluVx – Adj, AdjFluVx with Adj removed.

**Fig. 6.**
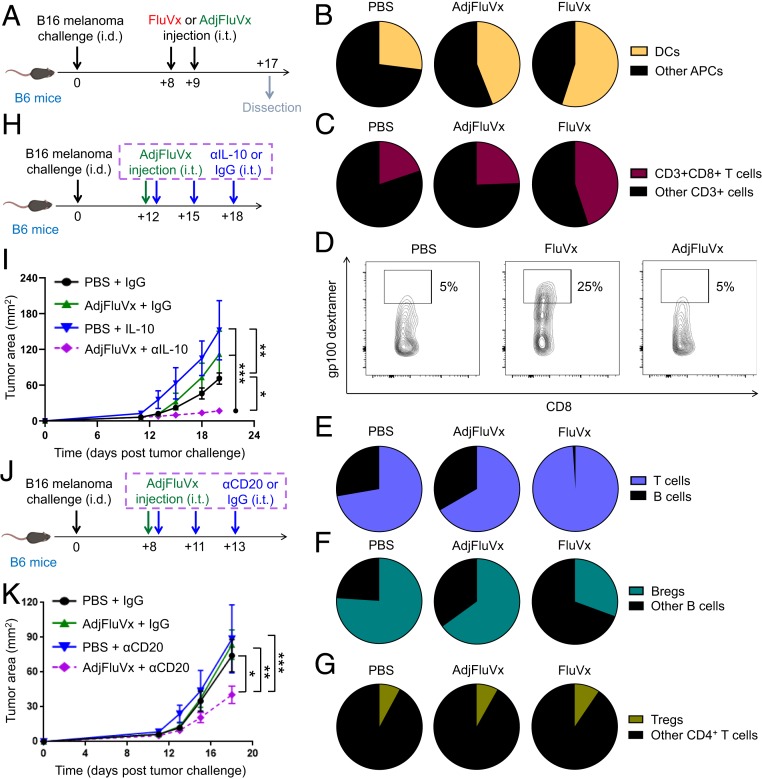
Intratumoral adjuvanted seasonal influenza vaccine administration fails to increase T cells but maintains regulatory B cells in the tumor. (*A*) Experimental design. Unadjuvanted seasonal influenza vaccine (FluVx): FluVx2. *n* = 3 to 5 pooled tumors/group. Data are representative of at least 2 independent experiments with similar results. (*B*) Cumulative pie charts of dendritic cells (CD11c^+^) among intratumoral APCs (CD45^+^MHC-II^+^) from experiment described in *A* and Fig. 4*D*. (*C*) Cumulative pie charts of CD8^+^ T cells (CD8^+^) among intratumoral T cells (CD45^+^CD3^+^) from experiment described in *A* and Fig. 4*D*. (*D*) Cumulative flow cytometry plots of tumor antigen-specific (gp100 dextramer^+^) CD8^+^ T cells among intratumoral CD8^+^ T cells (CD45^+^CD3^+^CD8^+^) from a similar experiment as described in *A*. Cumulative pie charts of B cell (CD19^+^) to T cell (CD3^+^) ratio among intratumoral T and B cells (CD45^+^CD3^+^CD19^−^ and CD45^+^CD19^+^CD3^−^) from experiment described in *A*. (*F*) Cumulative pie charts showing ratio of Bregs (IL-10^+^) among intratumoral B cells (CD45^+^CD20^+^) from experiment described in *A*. (*G*) Cumulative pie charts of Tregs (FOXP3^+^) among intratumoral CD4^+^ T cells (CD45^+^CD3^+^CD4^+^) from experiment described in *A*. (*H*) Experimental design. *n* = 3 to 4 mice/group. Data are representative of at least 2 independent experiments with similar results. (*I*) Tumor growth curves from experiment described in *H*. (*J*) Experimental design. *n* = 4 to 10 mice/group from 2 experiments with similar results. (*K*) Tumor growth curves from experiment described in *J*. *P < 0.05, **P < 0.01, ***P < 0.001 [2-way ANOVA with Tukey correction (*I* and *K*)]. Error bars: mean ± SEM. i.d., intradermal; i.t., intratumoral; FluVx, unadjuvanted seasonal influenza vaccine; AdjFluVx, adjuvanted seasonal influenza vaccine.

While all tested unadjuvanted influenza vaccines resulted in improved antitumor outcomes, an available adjuvanted formulation (hereafter referred to as AdjFluVx, *SI Appendix*, Table S1) demonstrated no tumor-reduction effect (Fig. 5 *A* and *B*). We recognized this difference between the unadjuvanted and adjuvanted formulations as a unique opportunity to uncover additional mechanisms that drive tumor regression versus progression. Importantly, AdjFluVx, which has been demonstrated in clinical trials to afford antiinfluenza virus protection (particularly in patients over 65 y old) (33, 34), did provide protection against active influenza virus even with intratumoral administration in our model (Fig. 5 *C* and *D*), demonstrating a disconnect between antitumor and antipathogen responses. Since the unique characteristic of AdjFluVx is its squalene-based adjuvant (35, 36), we sought to determine whether this adjuvant is responsible for AdjFluVx’s lack of antitumor efficacy. Although intratumoral injection of squalene-based adjuvant, AddaVax (Adj) (37–39), alone did not alter tumor growth, the addition of Adj to FluVx abrogated FluVx’s ability to reduce tumor growth (Fig. 5 *E* and *F*). Consistent with these tumor growth alterations, analysis of the full NanoString PanCancer Immune Profiling Panel demonstrated a decreased immune signaling signature with AdjFluVx (and with Adj added to FluVx [FluVx + Adj]) compared to FluVx (Fig. 5*G* and Dataset S3). Further, removing the adjuvant from AdjFluVx afforded it the ability to reduce tumor growth (Fig. 5 *H* and *I*). Although AdjFluVx increased the proportion of DCs among APCs in the tumor (albeit less than FluVx), AdjFluVx did not augment CD8^+^ T cells (including tumor antigen-specific T cells) within the tumor, ultimately failing to produce the elevated T cell:B cell ratio achieved by FluVx, and instead resulting in elevated influenza virus-specific antibodies in the tumor (Fig. 6 *A*–*E* and *SI Appendix*, Figs. S12 and S13). Further, we observed an increased proportion of intratumoral Bregs (IL-10–producing B cells) with AdjFluVx compared to FluVx administration, without an increase in Tregs (Fig. 6 *F* and *G* and *SI Appendix*, Fig. S14). Regulatory B cells have been associated with diminished antitumor immunity, and IL-10 produced by regulatory B cells can suppress CD8^+^ T cell functions, thereby abrogating their ability to mount a cytotoxic antitumor immune response (9, 40, 41). Importantly, intratumoral depletion of B cells or IL-10 blockade rendered AdjFluVx the ability to reduce tumor growth (Fig. 6 *H*–*K*).

## Discussion

Clinical successes utilizing immunotherapy to improve and prolong the lives of patients with cancer have demonstrated a vital role for the immune system in the treatment for cancer. However, thus far immunotherapies have been able to produce durable responses only in a limited proportion of patients. Therefore, to make the next great leap forward, innovative means of engaging the immune system are needed. In our described studies, we have focused on utilizing pathogens to augment inherently weaker antitumor immune responses to generate improved local and systemic cancer outcomes. Importantly, we observed that active influenza virus injection in the lung reduces tumor growth in the lung (even when a melanoma cell line was used that does not undergo productive infection by active influenza virus). However, active influenza virus injection in the skin did not reduce growth of that same melanoma cell line present in the skin. A difference between the lung and the skin is that the lung inherently contains natural cell targets for active influenza virus infection, while the skin does not. Active influenza A viruses bind to specific sialic acid residues on epithelial cells in the upper respiratory tract and subsequently gain entry into the cell and replicate, while cells in the skin lack the specific sialic acid residues necessary for productive influenza virus infection (42). Thus, in lung tissue, productive infection leads to a potent immune response to influenza virus by creating an immunologically inflamed hot microenvironment in the same tissue as the tumor. Without a major natural target for active influenza virus in the skin, the cells most likely to be affected are dendritic cells, which rather than boosting an immune response are dysregulated when active influenza virus is injected in the skin (28), further decreasing the ability of the tumor microenvironment to become immunologically hot.

In recent years, viral infection has been harnessed as a vehicle to augment antitumor immune responses, and in particular, oncolytic virus (OV) therapy has been employed as a tool in the clinic. Oncolytic viruses preferentially lyse tumor cells and consequently release tumor antigens and danger-associated molecular patterns (DAMPs) (43). However, in the context of oncolytic viruses, productive infection of the tumor cells themselves is the focus. In this setting, the overexpression of specific proteins by tumor cells (but not normal adjacent cells) is hijacked by oncolytic viruses, which use these overexpressed proteins as entry receptors or to facilitate their own replication. Normal cells with less expression of these proteins do not serve as the major target and are spared, or they utilize IFN signaling (a pathway that is dysregulated in cancer cells) to limit infection. Thus, a major focus in this field has centered on the importance of direct infection of the tumor cell as a prerequisite for generating antitumor immunity. However, the idea that tumor cell lysis by the pathogen is essential has been recently challenged by evidence demonstrating that an inactivated oncolytic virus is capable of initiating antitumor immunity via the STING pathway and may support better immunity than its active oncolytic virus counterpart (44). Additionally, our data indicate that TLR activation via interaction with viral-derived PAMPs is increased in the context of inactivated virus, which may initiate an innate immune response and thereby remodel the tumor microenvironment. Further in support of this is previous research demonstrating that pathogen vaccines can substitute for synthetic TLR agonists to stimulate dendritic cells (45). Our data demonstrate that inactivation of a nononcolytic virus, such as influenza, can augment an antitumor immune response when administered via intratumoral injection, even when the corresponding virus (in active form) is incapable of such activity (as in our setting of active influenza virus administration within a skin melanoma). This indicates that the field of microbial-based cancer therapies (MBCTs), which has experienced a recent resurgence of interest (46, 47), is not limited to the oncolytic class of pathogens or even to the use of active pathogens. Furthermore, in terms of clinical translation, inactivated influenza virus injection can be made available to immunosuppressed patients who are not eligible for active pathogen-based therapies and to patients concerned about sequalae that may result from active pathogen administration.

Studies have reported that pathogen-specific (e.g., cytomegalovirus [CMV], influenza virus, Epstein-Barr virus [EBV], etc.) CD8^+^ T cells infiltrate mouse and human tumors and comprise a significant fraction of intratumoral CD8^+^ T cells (48–52). The impact of such antiviral immune responders on antitumor immunity demands further investigation and may have important implications for the use of MBCTs in the clinic. Interestingly, patients whose tumors harbor the tetrapeptide, ESSA, a sequence shared by CMV, have been shown to exhibit increased survival in the context of CTLA-4 blockade (53). Further, recently in mouse models, virus-specific memory T cells have been shown to halt tumor growth when their cognate antigens are injected within the tumor to create an immune-“alarming” effect (49). In contrast to this strategy, which requires previous immunity against a specific pathogen, our work suggests that pathogen-related therapies can be harnessed for antitumor immune responses independent of previous exposure; as in the majority of our studies, the hosts had no previous exposure to influenza virus. However, even in such cases, intratumoral administration of inactivated influenza virus increases dendritic cells and antitumor CD8^+^ T cells and consequently the reduction of tumor growth, without any prerequisite immunity. This may be particularly important for repurposing the seasonal flu shot for cancer immunotherapy and translating it to clinical care, as the seasonal influenza vaccine includes antigens that are altered yearly to match the anticipated predominant strains of the upcoming season. In this context, our lack of the need for previous exposure to the same pathogen and strain is a major advantage. However, it is also important to note that in our studies, previous infection followed by resolution of a particular strain of influenza virus did not prohibit subsequent tumor reduction with intratumoral inactivated influenza virus (i.e., a vaccine) made from the exact same strain. This suggests that patients with or without previous immunity to the influenza virus strain contained within the utilized flu shot may benefit from intratumoral administration of the vaccine. With multiple strains included within each trivalent and quadrivalent flu shot, it may be that an optimal response is achieved when a combination of new and previously experienced antigens is utilized. In this scenario, previously experienced antigens quickly raise inflammatory immune responses, which inherently are likewise quickly quenched with the elimination of the recognized antigen. At the same time, new antigens raise slower responses that are maintained longer and may have sustained positive effects on antitumor immunity.

Our study proposes that intratumoral injection of an unadjuvanted seasonal influenza vaccine reduces tumor growth by converting immunologically inactive cold tumors to immune-infiltrated hot tumors, by augmenting DCs (including cross-presenting DCs) and tumor antigen-specific CD8^+^ T cells within the tumor microenvironment. These findings have important implications for the role of intratumoral seasonal influenza vaccination in priming patients to respond to existing immunotherapies (including, PD-1 and CTLA-4 blocking antibodies). Specifically, our study shows that intratumoral seasonal influenza vaccination 1) can reduce tumors on its own, 2) improves outcomes in the context of tumors that respond to PD-L1 therapy, 3) can reduce tumors even when they are resistant to PD-L1 blockade, and 4) in combination with PD-L1 blockade results in drastic reductions in tumor growth. This suggests that in patients, such vaccination may confer increased efficacy of immune-related therapies, including checkpoint blockade treatments that have dramatically improved survival for a segment of the cancer patient population, but which have not yet been made effective for all patients with cancer (54).

Important attention must be paid to the formulation of the vaccine, as some adjuvants may provide improved antipathogen protection, while limiting the ability of the vaccine to improve antitumor outcomes. The adjuvanted seasonal influenza vaccine utilized in our studies has been demonstrated to provide enhanced immunity against active influenza lung infection in persons 65 y of age and older, whose immunity may decrease with increasing age (33, 34). However, this adjuvanted formulation does not augment an antitumor immune response when administered via intratumoral injection, but instead maintains immunosuppressive regulatory B cells within the tumor. Adjuvants play an important role in boosting immune responses. Likely within our unadjuvanted seasonal influenza vaccines natural adjuvants (e.g., host cell proteins and DNA, residual influenza ssRNA, etc.) resulting from the process of manufacturing the inactivated seasonal influenza vaccine likewise improve immunity by interacting with multiple danger-sensing mechanisms (e.g., an RNA-sensing toll-like receptor), an interaction that has been previously shown to improve antitumor immune responses (55). Since the majority of seasonal influenza vaccines currently on the market are inactivated and do not contain manufactured adjuvants (e.g., squalene), and such vaccines have a high safety profile and are FDA approved, the translatability of these as innovative immunotherapies for cancer is high and the barriers to reaching many patients with cancer is low.

Although active influenza virus lung infection is a major public health concern, with tens of thousands of deaths documented annually in the United States (56), the Centers for Disease Control and Prevention (CDC) has reported that in the 2017–18 season, only 37.1% of adults received the seasonal influenza vaccine (57). Anecdotally, this percent may be even lower among patients with cancer, in whom infections have also been reported to have greater morbidity and mortality. Importantly, beyond demonstrating that influenza vaccination administered via intratumoral injection can reduce tumor growth, our studies provide evidence that protection against future active influenza lung infection can be provided via intratumoral administration. This suggests that patients receiving intratumoral seasonal influenza vaccination may experience multiple clinical benefits and that seasonal influenza vaccination is a crucial public health tool that may be utilized as both a preventive measure against infection and an immunotherapy for cancer.

## Materials and Methods

### Mice.

Mice were housed in specific-pathogen-free facilities and all experiments were conducted in accordance with procedures approved by the Institutional Animal Care and Use Committee (IACUC) and Institutional Biosafety Committee at Rutgers, The State University of New Jersey and Rush University Medical Center, and the Institutional Review Board at Rutgers, The State University of New Jersey. B6 (C57BL/6J), Batf3^−/−^ (B6.129S(C)-*Batf3*^*tm1Kmm*^/J), NSG (NOD.Cg-*Prkdc*^*scid*^*Il2rg*^*tm1Wjl*^*/SzJ;* NOD SCID gamma), and BALB/c mice were purchased from The Jackson Laboratory at 6 to 10 wk of age.

### Active Influenza and Heat-Inactivated Influenza Virus.

For experiments utilizing active influenza virus infections, mice were administered 1 × 10^6^ plaque-forming units (pfu) of A/PR8/1934/H1N1 (FLU) (58) or OVA_257–264_ SIINFEKL-expressing A/PR8/1934/H1N1 (FLU-OVA) (59) by passive i.n. or i.t. (i.e., at the tumor site) administration (25 to 50 μL). Control mice were administered an equal volume of phosphate-buffered saline (PBS) via the same route. For experiments utilizing heat-inactivated influenza virus (hiFLU or hiFLU-OVA), the virus was inactivated by incubating active A/PR8/1934/H1N1 FLU at 90 °C for 5 min on an IncuBlock Plus heat block (Denville Scientific) prior to injection into mice. For experiments utilizing influenza virus lysate (FLU lysate), active A/PR8/1934/H1N1 FLU was resuspended in RLT buffer (Qiagen) for 1 h to generate a lysate. RLT buffer was then dialyzed using a Slide-A-Lyzer G2 Dialysis Cassette (10-kDa molecular weight cutoff; Thermo Fisher) prior to lysate administration.

### Vaccines and Adjuvants.

FDA-approved 2017–2018 seasonal influenza vaccines were purchased from their respective manufacturers: FLUCELVAX (FluVx1; Seqirus), FLUVIRIN (FluVx2; Seqirus), FLUARIX QUADRIVALENT (FluVx3; GlaxoSmithKline), FLUBLOK (FluVx4; Protein Sciences Corporation), and FLUAD (AdjFluVx; Seqirus). Vaccine details are provided in *SI Appendix*, Table S1. To mimic adjuvant MF59 (Novartis), AddaVax (Adj; Invivogen) was administered via intratumoral injection (50 μL). Control mice were administered PBS at the same volume via the same route. In experiments in which the adjuvant and vaccine were jointly delivered, 50 µL adjuvant + 50 µL vaccine were mixed and delivered in a total volume of 100 µL via intratumoral injection. In some experiments, MF59, which is primarily composed of squalene, was removed by centrifugal filtration using Amicon Ultra centrifugal filter units with regenerated cellulose filters (with a 30-kDa molecular weight cutoff). MF59-containing AdjFluVx (500 μL) was added to the unit and washed with acetone (250 μL; 3 times) followed by PBS (250 μL; 3 times). The protein component of the vaccine was collected using a pipette, freeze dried, and reconstituted to the original volume using PBS.

### Tumor Challenge.

For tumor challenge experiments, B6 and NSG mice were anesthetized with isoflurane and administered 100,000 to 150,000 B16-F10 melanoma cells (American Type Culture Collection [ATCC]) via i.v. or intradermal (i.d.) injection and BALB/c mice were anesthetized with isoflurane and administered 100,000 to 150,000 4T1 triple-negative breast cancer cells (ATCC) in the mammary fat pad. B16-F10 and 4T1 cancer cell lines were cultured in Dulbecco’s Modified Eagle Medium (Gibco), 10% fatal bovine serum (Sigma-Aldrich), 100 units/mL penicillin (Gibco), 100 mg/mL streptomycin (Gibco), and 0.29 mg/mL glutamine (Gibco) prior to harvesting for tumor injection. Primary tumor growth was monitored by Vernier caliper measurements in 2 perpendicular directions serially after tumor challenge. Mice harboring tumors were killed when the tumor area reached 20 mm in any direction or met other health-related endpoints, as per institutional IACUC policies. To quantify 4T1 lung metastases, 5% India ink (Fisher Scientific) diluted in distilled water was injected into the trachea after euthanasia (60). Lungs were dissected and transferred to Fekete’s solution (40 mL glacial acetic acid, 32 mL [37%] formalin, 700 mL 100% ethanol, and 228 mL double-distilled water) and washed 3 to 4 times in this solution and once in PBS. 4T1 lung surface metastases (white in appearance) and B16-F10 lung surface foci (black in appearance) were manually counted with the use of a magnifying glass.

### Statistical Analyses.

Two-tailed Student *t* test (for 2 groups) or 1-way ANOVA with Tukey correction (for more than 2 groups) was used to determine statistical significance for data comparisons at a single timepoint. Two-way ANOVA or mixed-effects model with Bonferroni (for 2 groups) or Tukey (for more than 2 groups) correction was used to determine statistical significance for data comparisons with multiple timepoints. Kruskal–Wallis with Dunn’s multiple comparisons test was performed for focused comparisons of 1 group to all other groups at a single timepoint. Mantel–Cox log rank test was performed to determine statistical significance for the comparison of survival curves. Prism version 8.0 (GraphPad) was used for generation of all graphs and performance of statistical and Extreme Studentized Deviate analyses, except for Fig. 1 *C* and *D*, where STATA version 15.0 (StataCorp, LLC) was used to perform statistical analyses. Statistical significance shown for survival curves represents a comparison of the 2 survival curves. Statistical significance shown for all other graphs represents comparisons at the indicated timepoint. Statistical significance is denoted as ns, not significant, *P < 0.05, **P < 0.01, and ***P < 0.001. Comparisons with significance at *P* < 0.001 or *P* < 0.0001 are listed as ***P < 0.001.

### Data Availability.

Sequencing data are included in the supplementary materials for this manuscript: Datasets S1–S3.

## Supplementary Material

Supplementary File

Supplementary File

Supplementary File

Supplementary File
