# Supplementary material for: Intratumoral injection of the seasonal flu shot converts immunologically cold tumors to hot and serves as an immunotherapy for cancer
Source: Proc Natl Acad Sci U S A. 2019 Dec 30;117(2):1119–28. doi: 10.1073/pnas.1904022116 (PMC6969546; doi:10.1073/pnas.1904022116)
Supplement: Supplementary File [file pnas.1904022116.sapp.pdf]

PNAS
www.pnas.org

Supplementary Information for

Intratumoral Injection of the Seasonal Flu Shot Converts Immunologically Cold Tumors to Hot and Serves as an Immunotherapy for Cancer

Jenna H. Newman, C. Brent Chesson, Nora L. Herzoga, Praveen K. Bommareddy, Salvatore M. Aspromonte, Russell Pepe, Ricardo Estupinian, Mones M. Aboelatta, Stuti Buddhadev, Saeed Tarabichi, Michael Lee, Shengguo Li, Daniel J. Medina, Eileena F. Giurini, Kajal H. Gupta, Gabriel Guevara-Aleman, Marco Rossi, Christina Nowicki, Abdulkareem Abed, Josef W. Goldufsky, Joseph R. Broucek, Raquel E. Redondo, David Rotter, Sachin R. Jhawar, Shang-Jui Wang, Frederick J. Kohlhapp, Howard L. Kaufman, Paul G. Thomas, Vineet Gupta, Timothy M. Kuzel, Jochen Reiser, Joyce Paras, Michael P. Kane, Eric A. Singer, Jyoti Malhotra, Lisa K. Denzin, Derek B. Sant'Angelo, Arnold B. Rabson, Leonard Y. Lee, Ahmed Lasfar, John Langenfeld, Jason M. Schenkel, Mary Jo Fidler, Emily S. Ruiz, Amanda L. Marzo, Jai S. Rudra, Ann W. Silk, and Andrew Zloza

Corresponding author: Andrew Zloza
Email: andrew_zloza@rush.edu

This PDF file includes:

SI Materials and Methods
Figures S1 to S14
Table S1
SI References

Other supplementary materials for this manuscript include the following:

Datasets S1 to S3

www.pnas.org/cgi/doi/10.1073/pnas.1904022116

SI Materials and Methods

SEER-Medicare linked database subjects

Study subjects were identified from the SEER-Medicare Linked Database and SAS version 9.4 (SAS Institute, Inc.) was used to analyze these data. All cases of primary stage I to II non-small cell lung cancer (NSCLC; tumor site codes 34.0-34.9 and ICD-O-2 morphology codes 8010-8040, 8050-8076, 8140, 8143, 8250-8260, 8310, 8320, 8323, 8470-8490, and 8550-8573), age > 65 years, treated with either surgery or chemo-radiation during the span of a 100 months between 2001 and 2011 were included. Samples were limited to patients with histologically confirmed cancers and excluded cases diagnosed at autopsy or death certificate. Survival was determined as the interval from the date of cancer diagnosis to the Medicare date of death. These data are updated daily by Medicare and thus are current as of the day that data were extracted for linkage with SEER. For analyses involving lung-cancer specific survival, SEER survival data were used as Medicare does not provide information regarding the cause of death. Data on the cause of death in SEER were obtained from state death certificates and included in the PEDSF file using ICD-9 codes. Presence of influenza virus infection was considered 'yes' (FLU dx) based on codes obtained from MEDPAR files for hospitalization for influenza virus infection. Since lung cancer-specific mortality did not reach 50% for the FLU dx group, time to mortality (lung cancer-specific and overall) in 25% of the patients (P25) was calculated.

Autologous immune-reconstituted patient-derived xenograft (AIR-PDX) mouse model

Immune-deficient NSG mice were purchased from Jackson Laboratory and bred in-house. Patient-derived tumor tissue and peripheral blood were obtained with patient consent and under IRB approval through the Rutgers Cancer Institute of New Jersey Biospecimen Repository and Histopathology Service, which serves as an "honest broker" and maintains the chain of custody for patient samples made available to investigators at Rutgers Cancer Institute of New Jersey. Two sets of AIR-PDX mice were created, one set from a patient whose de-identified clinical annotation described the tissue as a primary lung tumor and one from a patient whose clinical annotation defined the tissue as a melanoma metastasis to lymph node. AIR-PDX mice were created within

24 hours of obtaining autologous (*i.e.*, from the same patient) tumor tissue and peripheral blood. Specifically, male and female NSG mice (>8 weeks of age) received adoptive transfer (via intraperitoneal injection [i.p]) of fresh peripheral blood mononuclear cells (PBMCs) after extraction from peripheral blood by Ficoll gradient centrifugation. PBMCs were washed and re-suspended in PBS for injection (500,000 cells in 100 µL per mouse). Additionally, these NSG mice were surgically implanted with fresh tumor (~5 x 5 x 3 mm tumor sections; one per mouse) into a 5-mm incision made in the right flank and closed with a 6-0 proline horizontal mattress suture (Henry Schein). Mice recovered from this minor survival surgery in warming cages and were monitored for assurance of a minimum respiratory rate of 30 breaths/minute. Within two weeks of adoptive transfer and surgery, AIR-PDX mice were surveyed for successful tumor implantation (defined as stable or increasing tumor size compared to implantation size). Subsequently, AIR-PDX mice created from a patient-derived primary lung tumor received intratumoral FluVx (FluVx1) or PBS on day 0 (*i.e.*, the first day of treatment), and AIR-PDX mice created from a patient-derived melanoma lymph node metastasis received intratumoral FluVx (FluVx1) or PBS on days 0 and 2.

Depletions, blockade, and adoptive cell transfer

In vivo antibody-mediated depletions and blockades were performed using the following antibodies: αCD20 (BioLegend, clone SA271G2), αCD8 (BioXCell, clone 2.43), αIL-10 (BioXCell, clone JES5-2A5), and αPD-L1 (BioXCell, clone 10F.9G2), or their respective isotype control antibodies. Antibodies were diluted to desired concentrations in *InVivo*Pure pH 6.5 Dilution Buffer (BioXCell) and administered at either 250 µg via intraperitoneal injection or 50 µg via intratumoral injection. In experiments requiring transfer of splenic cells from donor to recipient mice, spleens were mechanically dissociated through a 70-µm filter and red blood cells were removed using 1 mL of Ack Lysing Buffer (Gibco) per spleen under sterile conditions. Cells were washed and re-suspended in PBS and adoptively transferred to recipient mice (~5x10^7) via intraperitoneal (i.p.) injection in a total volume of 100 µL.

Tissue processing and flow cytometry

For optimal staining of IL-10, 500 µg of monensin (Sigma-Aldrich) dissolved in dimethyl sulfoxide (DMSO) (Sigma Aldrich) was administered via i.v. injection (retro-orbital sinus) into mice six hours prior to euthanasia and dissection. Resected tumors were mechanically dissociated using a gentleMACS™ Octo Dissociator utilizing program Tumor 01_01 (Miltenyi Biotec) in HBSS, enzymatically dissociated in HBSS containing 1 mg/mL type IV collagenase from *Clostridium histolyticum* (Sigma-Aldrich) and 40 µg/mL DNAse I from bovine pancreas (Sigma-Aldrich) at 37°C for 30 minutes with constant rocking, and then mechanically dissociated using Dissociator program Tumor 02_01. Dissociated cells were applied to a 70-µm filter and subsequently washed with PBS to yield a single-cell suspension. Spleens were mechanically dissociated through a 70-µm filter and red blood cells were removed using 1 mL of Ack Lysing Buffer per spleen. Cells were washed with PBS prior to staining for flow cytometry. H-2Db gp100 dextramer, KVPRNQDWL (Immudex), and H-2Kb TRP2 dextramer, SVYDFFVWL (Immudex), were applied to cells at a concentration of 5 µL/dextramer in a total volume of 50 µL PBS for 20 minutes at room temperature. Extracellular staining was subsequently performed using antibodies specific to CD3, CD4, CD8, CD11c, CD19, CD20, CD45, H-2Kb-SIINFEKL ($OVA_{257-264}$), and MHC-II, purchased from either BioLegend or eBiosciences/ThermoFisher Scientific, using 1-5 µL/test in a total volume of 100 µL PBS for 30 minutes at room temperature. The LIVE/DEAD Fixable Aqua Dead Cell Stain Kit for 450 nm excitation (ThermoFisher Scientific) at 0.25 µL/test was added to each sample. Cells were washed with PBS before proceeding to intracellular staining steps. Intracellular staining was performed using the True-Nuclear™ Transcription Buffer Set (BioLegend), in accordance with manufacturer's protocol. Antibodies selective for FOXP3 and IL-10 (eBiosciences/ThermoFisher Scientific) were used at 1-5 µL/test in a total of 100 µL of 1X Perm Buffer (BioLegend). Cells were washed three times in Perm Buffer and fixed using BD™ Stabilizing Fixative (Becton Dickinson). Flow cytometry was performed using a BD LSR II cytometer. Analysis of flow cytometry was performed using Flow Jo (TreeStar, version 10). Flow plots shown have undergone gating on live singlet lymphocytes utilizing forward scatter (FSC) and side scatter (SSC) height, area, and width, as well as LIVE/DEAD, as previously described (1).

Quantitative PCR and NanoString analysis

Dissected tumors and lungs were stored in 1mL of TRIzol™ (Invitrogen) at -80°C until RNA extraction was performed. Subsequently, tissues were homogenized using Benchmark's BEADBUG™ 6 Microtube Homogenizer. RNA was isolated using the Qiagen RNeasy Plus Mini Kit. The purity of the resulting RNA was measured using a NanoDrop™ spectrophotometer (Thermo Fisher Scientific). Quantitative reverse transcription polymerase chain reaction (qRT-PCR) for influenza virus was performed using the primers 5' CATGGAATGGCTAAAGACAAGACC (forward), 5' CCATTAAGGGCATTTTGGACA (reverse), and the TaqMan® probe FAM- 5′ TTTGTGTTCACGCTCACCGTGCCCATAMRA (ThermoFisher Scientific). GAPDH was used as a housekeeping gene control. qRT-PCR was conducted using a StepOnePlus Real-Time PCR System (Applied Biosystems). Profiling of transcripts implicated in the anti-tumor immune response was assessed via the NanoString PanCancer Immune Profiling Panel using an nCounter Digital Analyzer (NanoString Technologies). Total RNA (100 ng) was used for each sample analyzed. Analysis of NanoString data was performed using nSolver Analysis software.

T cell receptor (TCR) sequencing and analysis

Tumors were frozen at -80ºC upon dissection. Samples were analyzed by Adaptive Biotechnologies utilizing the immunoSEQ® assay, which assesses diversity and clonality of the CDR3 region of the TCR of T cells. Sequences were characterized by utilizing a multiplex PCR strategy followed by Illumina sequencing (2-6). Data were analyzed using immunoSEQ® Analyzer software and Excel (Microsoft). To determine whether TCR clones within the tumor microenvironment of a control (PBS-injected) tumor are expanded with FluVx treatment (*i.e.*, demonstrate increased clonality/evenness), the mean representation of tumor-associated TCR clones was analyzed. Productive TCR clones were derived from FluVx1-treated and control tumors and compared. TCR clones from control tumors were considered "tumor-associated." Tumor-associated TCR clones represented in at least one control tumor (n) and detected in a greater number of FluVx1-treated tumors (at least n + 1) were further considered. Among these TCR

clones, average clonality within control tumors was compared to FluVx1-treated tumors and a graph was generated.

Influenza virus antibody ELISA

Nunc MaxiSorp flat-bottom plates (ThermoFisher Scientific) were coated with either FluVx1 or AdjFluVx at a concentration of 1 µg/mL diluted in PBST to a total volume per well of 100 µL. As a negative control, select wells were incubated with 100 µL of PBS without vaccine. After overnight incubation, the coating solution was discarded, and wells were washed once with 100 µL PBST buffer (PBS buffer with 0.05% Tween20). Plates were then blocked with 5% milk powder in PBST at 100 µL/well for one hour. Wells were subsequently washed three times with 100 µL PBST. Then, tumor homogenates were added to the wells at 1:1000 dilution with PBST at a final volume of 100 µL and incubated for one hour. Plates were subsequently washed five times in 100 µL PBST and then 100 µL TMB substrate was added to each well for three minutes. Next, 50 µL of stop solution (H_2SO_4) was added to each well. Contents of each well were transferred to a new plate and absorbance was read at 450 nm. Absorbance for each experimental well was calculated by subtracting background absorbance (derived from coated control wells in which PBST was added in place of tumor homogenate).

Toll-like receptor (TLR) signaling assay

The HEK-Blue-mTLR7 cell line (Invivogen), engineered to express mouse TLR7 (mTLR7), and its parental cell line, Null2-k (Invivogen), which does not express any mouse TLRs, were propagated and utilized to determine TLR7 activity, as per the manufacturer's instructions. In HEK-Blue-mTLR7 cells, secreted embryonic alkaline phosphatase (SEAP) is produced upon stimulation with a TLR7 ligand and real-time detection of SEAP is conducted utilizing HEK-Blue Detection medium (Invivogen) (7). Briefly, treatments and controls were plated in triplicate: 20 µl of active (MOI = 1) or heat-inactivated influenza A/PR8/1934/H1N1 virus, positive control TLR7 agonist, CL264 (50 µg/ml, Invivogen), and negative control, PBS (1x). Then, the respective cell lines (5×10^4 cells in

180 µl of medium per well) were added to the appropriate wells. The plate was incubated for 24 hours at 37^oC and 5% CO_2, and analyzed using a Cytation 3 (Biotek) plate reader at $OD_{620\ nm}$.

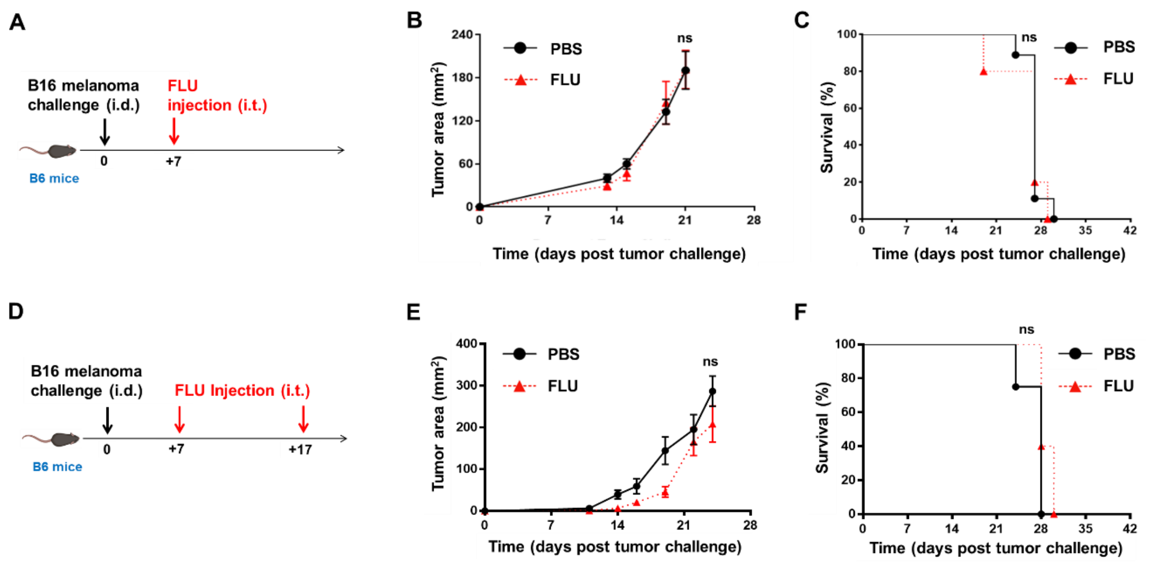

Fig. S1. Intratumoral active influenza virus administration does not reduce melanoma growth in the skin or prolong host survival. (*A*) Experimental design. n = 4-9 mice/group. Data are representative of at least two independent experiments with similar results. (*B*) Tumor growth curves from experiment described in (*A*). (*C*) Survival curves from experiment described in (*A*). (*D*) Experimental design, from experiment shown in Fig. 2*A*. n = 4-5 mice/group. Data are representative of at least two independent experiments with similar results. (*E*) Tumor growth curves from experiment described in (*D*). (*F*) Survival curves from experiment described in (*D*). ns, not significant [Two-way ANOVA with Bonferroni correction (*B, E*), Mantel-Cox log rank test (*C, F*)]. Error bars: mean ± s.e.m. i.d., intradermal. i.t., intratumoral. PBS, phosphate-buffered saline. FLU, active influenza virus.

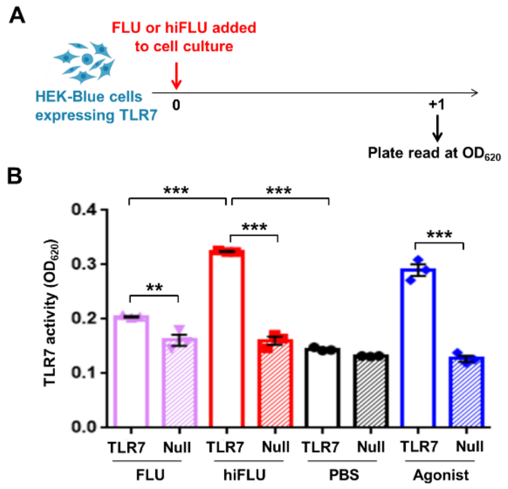

Fig. S2. Heat-inactivated influenza virus administration increases TLR7 activity to a greater extent than active influenza virus administration. (*A*) Experimental design. Data are from one experiment run in triplicate wells and representative of at least two independent experiments with similar results. (*B*) TLR7 activity read at $OD_{620\ nm}$ from experiment described in (*A*). ***P* < 0.01, ****P* < 0.001 [One-way ANOVA with Tukey correction]. Error bars: mean ± s.e.m. TLR7: HEK-Blue-mTLR7 cell line expressing mouse TLR7. Null: parental HEK293 cell line not expressing TLR7. FLU, active influenza virus. hiFLU, heat-inactivated influenza virus. PBS, phosphate-buffered saline. Agonist: TLR 7 agonist, CL264.

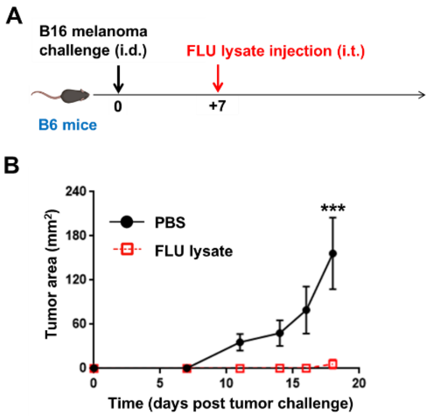

Fig. S3. Intratumoral influenza virus lysate administration reduces tumor growth in the skin. (*A*) Experimental design. n = 5 mice/group. Data are representative of at least two independent experiments with similar results. (*B*) Tumor growth curves from experiment described in (*A*). ****P* < 0.001 [Two-way ANOVA with Bonferroni correction]. Error bar: mean ± s.e.m. FLU lysate, influenza virus lysate. i.d., intradermal. i.t., intratumoral. PBS, phosphate-buffered saline.

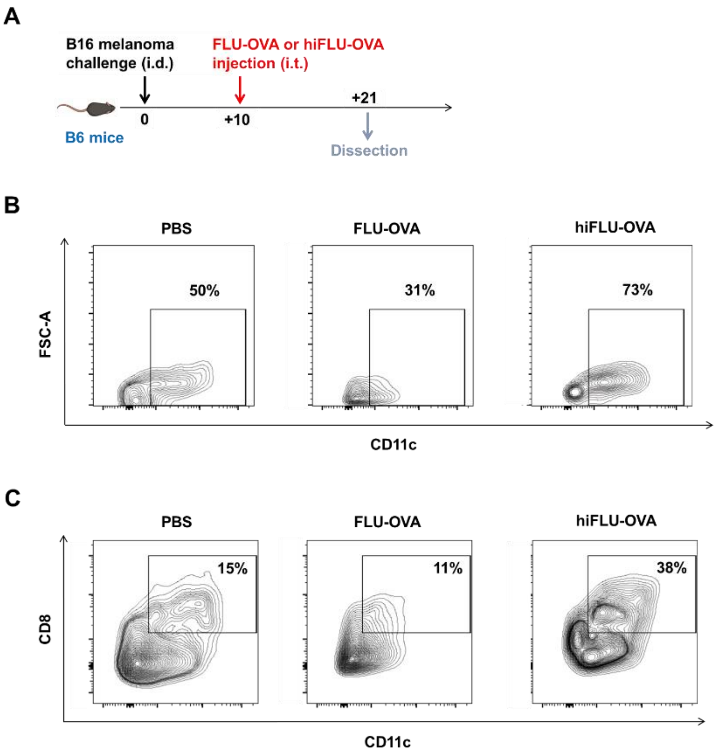

Fig. S4. Cumulative flow cytometry plots of intratumoral dendritic cells (DCs) from mice treated with FLU-OVA or hiFLU-OVA from experiment described in Figure 2*D-F*. (*A*) Experimental design. n = 3-5 pooled tumors/group. Data are representative of at least two independent experiments with similar results. (*B*) Cumulative flow cytometry plots of DCs ($CD11c^+$) among intratumoral antigen-presenting cells (APCs; $CD45^+$MHC-II^+). (*C*) Cumulative flow cytometry plots of cross-presenting DCs ($CD11c^+CD8^+$) among intratumoral APCs ($CD45^+$MHC-II^+). i.d., intradermal. i.t., intratumoral. PBS, phosphate-buffered saline. FLU-OVA, active influenza virus expressing SIINFEKL peptide from ovalbumin ($OVA_{257-264}$). hiFLU-OVA, heat-inactivated influenza virus expressing SIINFEKL peptide from ovalbumin ($OVA_{257-264}$). FSC-A, forward scatter area.

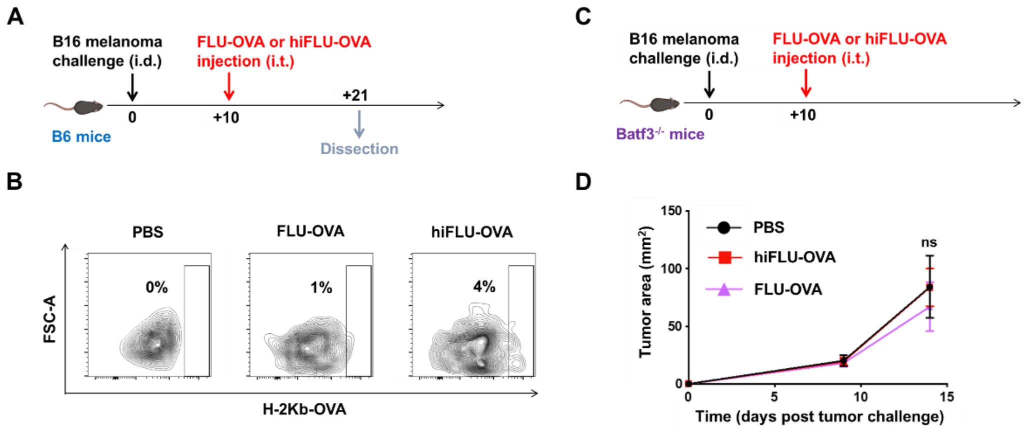

Fig. S5. Intratumoral heat-inactivated influenza virus administration increases antigen presentation by dendritic cells (DCs) and requires cross-presenting DCs for tumor growth reduction. (*A*) Experimental design. n = 3-5 pooled tumors/group. Data are representative of at least two independent experiments with similar results. (*B*) Cumulative flow cytometry plots of DCs presenting $OVA_{257\text{-}264}$ antigen (SIINFEKL) within H-2Kb MHC-I molecule, H-2Kb-OVA (H-2Kb-OVA^{+}) among intratumoral DCs ($CD45^{+}$MHC-$II^{+}$$CD11c^{+}$) from experiment described in (*A*). (*C*) Experimental design. n = 5 mice/group. (*D*) Tumor growth curves from experiment described in (*C*). ns, not significant [Two-way ANOVA with Tukey correction]. Error bars: mean ± s.e.m. i.d., intradermal. i.t., intratumoral. PBS, phosphate-buffered saline. FLU-OVA, active influenza virus expressing SIINFEKL peptide from ovalbumin ($OVA_{257\text{-}264}$). hiFLU-OVA, heat-inactivated influenza virus expressing SIINFEKL peptide from ovalbumin ($OVA_{257\text{-}264}$). FSC-A, forward scatter area.

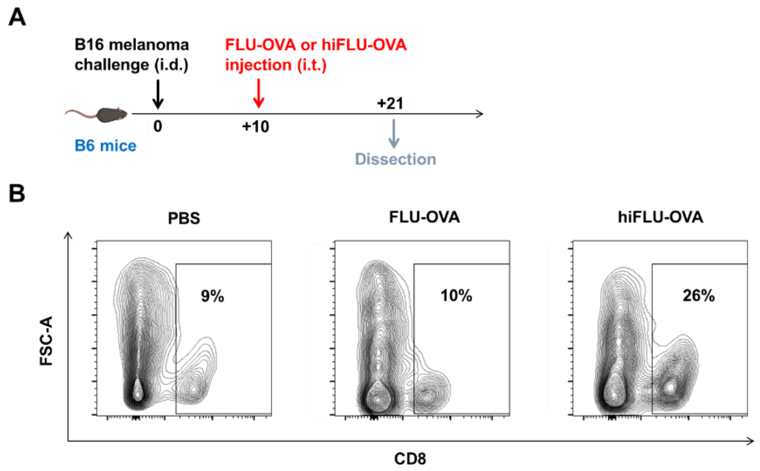

Fig. S6. Cumulative flow cytometry plots of intratumoral CD8+ T cells from mice treated with FLU-OVA or hiFLU-OVA, from experiment described in Figure 2*D* and *G*. (*A*) Experimental design. n = 3-5 pooled tumors/group. Data are representative of at least two independent experiments with similar results. (*B*) Cumulative flow cytometry plots of CD8$^+$ T cells (CD8$^+$) among intratumoral T cells (CD45$^+$CD3$^+$) for experiment descried in (*A*). i.d., intradermal. i.t., intratumoral. PBS, phosphate-buffered saline. FLU-OVA, active influenza virus expressing SIINFEKL peptide from ovalbumin (OVA$_{257\text{-}264}$). hiFLU-OVA, heat-inactivated influenza virus expressing SIINFEKL peptide from ovalbumin (OVA$_{257\text{-}264}$). FSC-A, forward scatter area.

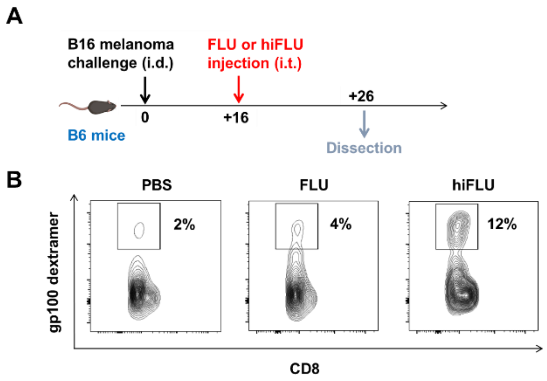

Fig. S7. Cumulative flow cytometry plots of intratumoral tumor antigen-specific CD8+ T cells from mice treated with FLU or hiFLU without $OVA_{257\text{-}264}$ peptide. (*A*) Experimental design. n = 3-5 pooled tumors/group. (*B*) Cumulative flow cytometry plots of tumor antigen-specific (gp100-dextramer^{+}) CD8^{+} T cells among intratumoral CD8^{+} T cells from the experiment described in (*A*). i.d., intradermal. i.t., intratumoral. PBS, phosphate-buffered saline. FLU, active influenza virus. hiFLU, heat-inactivated influenza virus.

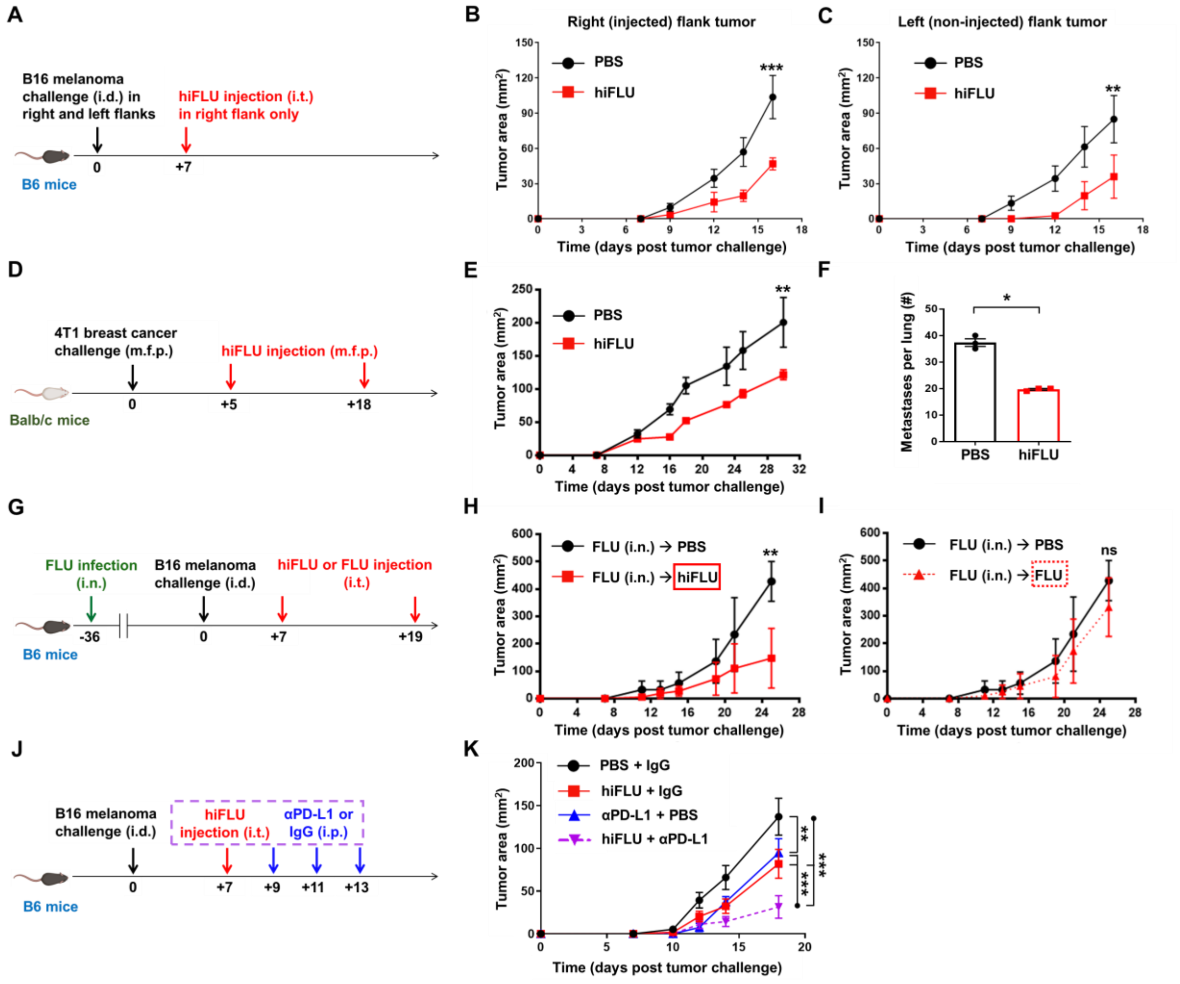

Fig. S8. Intratumoral heat-inactivated influenza virus administration reduces tumor growth systemically and in hosts previously infected with active influenza virus and augments responses to checkpoint blockade immunotherapy. (*A*) Experimental design. n = 5-12 mice per group. (*B*) Tumor growth curves of right (injected) flank tumors from experiment described in (*A*). (*C*) Tumor growth curves of left (non-injected) flank tumors from experiment described in (*A*). (*D*) Experimental design. n = 3-4 mice per group. (*E*) Tumor growth curves from experiment described in (*D*). (*F*) Bar graphs showing number of metastases per lung surface from experiment described in (*D*). (*G*) Experimental design. n = 3 mice per group. (*H*) Tumor growth curves for mice infected with active influenza virus and subsequently administered hiFLU at the tumor site from experiment described in (*G*). (*I*) Tumor growth curves for mice infected with active influenza virus and subsequently administered FLU at the tumor site from experiment described in (*G*). (*J*) Experimental design. n = 8-10 mice per group pooled from two similar experiments. (*K*) Tumor growth curves from experiments described in (*J*). ns, not significant, **P* < 0.05, ***P* < 0.01, ****P* < 0.001 [Two-way ANOVA with Bonferroni correction (*B, C, E, H, and I*) or Tukey correction (*K*), two-tailed student t test (*F*)]. Error bars: mean ± s.e.m. i.d., intradermal. i.t., intratumoral. m.f.p., mammary fat pad. i.n., intranasal. i.p., intraperitoneal. PBS, phosphate-buffered saline. FLU, active influenza virus. hiFLU, heat-inactivated influenza virus. IgG, control isotype antibody. αPD-L1, PD-L1 blocking antibody.

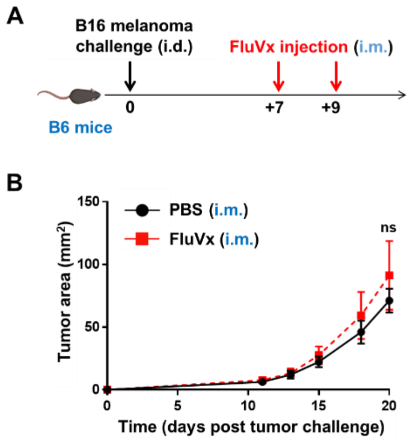

Fig. S9. Intramuscular unadjuvanted seasonal influenza vaccine administration does not reduce tumor growth in the skin. (*A*) Experimental design for intramuscular (i.m.) injection in the left lower limb). n = 4-5 mice/group. Unadjuvanted seasonal influenza vaccine (FluVx): FluVx2. Data are representative of at least two independent experiments with similar results. (*B*) Tumor growth curves from experiment described in (*A*). ns = not significant [Two-way ANOVA with Bonferroni correction]. Error bars: mean ± s.e.m. i.d., intradermal. PBS, phosphate-buffered saline.

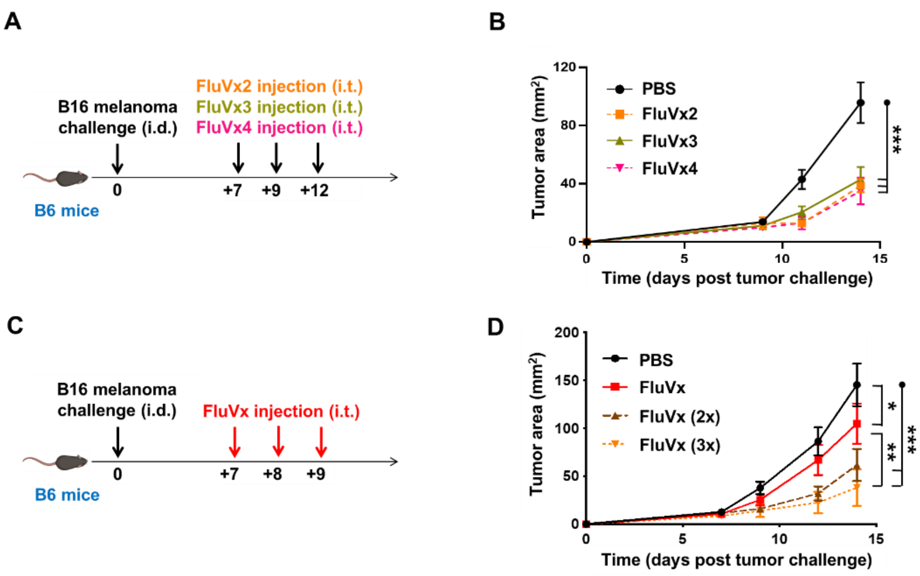

Fig. S10. Multiple 2017-2018 unadjuvanted seasonal influenza vaccines reduce tumor growth and intratumoral unadjuvanted seasonal influenza vaccination exhibits increased efficacy with multiple injections. (*A*) Experimental design for testing different unadjuvanted formulations of the seasonal influenza vaccine (FluVx): FluVx2, FluVx3, and FluVx4. These vaccines are defined in *SI Appendix* Table S1. n = 4-5 mice / group. (*B*) Tumor growth curves from experiment described in (*A*). (*C*) Experimental design for studies utilizing one [day 7; 1x], two [days 7 and 8; 2x], or three [days 7, 8 and 9; 3x] injections. n = 5-9 mice/group. FluVx: FluVx1. (*D*) Tumor growth curves from experiment described in (*C*). **P* < 0.05, ***P* < 0.01, ****P* < 0.001 [Two-way ANOVA with Tukey correction (*B* and *D*)]. Error bars: mean ± s.e.m. i.d., intradermal. i.t., intratumoral. PBS, phosphate-buffered saline.

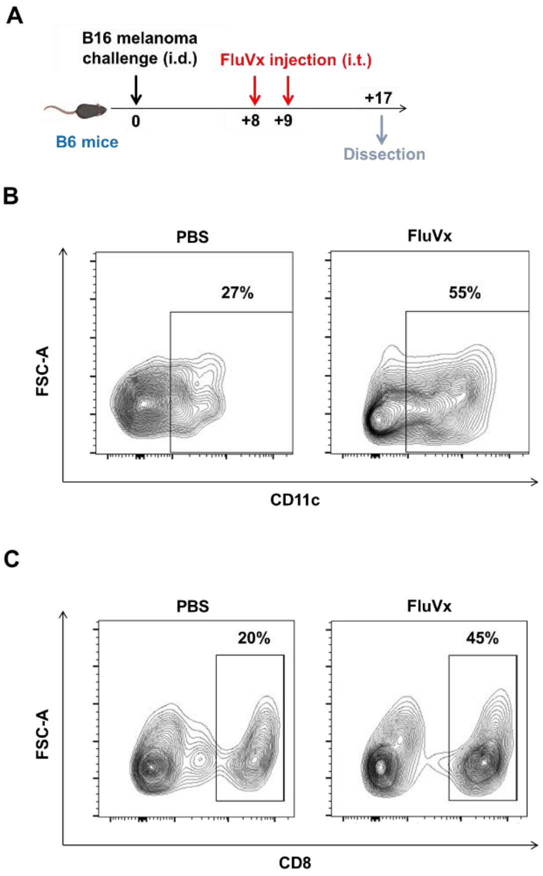

Fig. S11. Cumulative flow cytometry plots of intratumoral dendritic cells (DCs) and CD8+ T cells from mice treated with unadjuvanted seasonal influenza vaccine, from experiment described in Figure 4*D-F*. (*A*) Experimental design. n = 3-5 pooled tumors/group. Unadjuvanted seasonal influenza vaccine (FluVx): FluVx1. Data are representative of at least two independent experiments with similar results. (*B*) Cumulative flow cytometry plots of DCs ($CD11c^+$) among intratumoral antigen-presenting cells (APCs; $CD45^+MHC\text{-}II^+$) from experiment described in (*A*). (*C*) Cumulative flow cytometry plots of CD8+ T cells ($CD8^+$) among intratumoral T cells ($CD45^+CD3^+$) from experiment described in (*A*). i.d., intradermal, i.t., intratumoral. PBS, phosphate-buffered saline. FSC-A, forward scatter area.

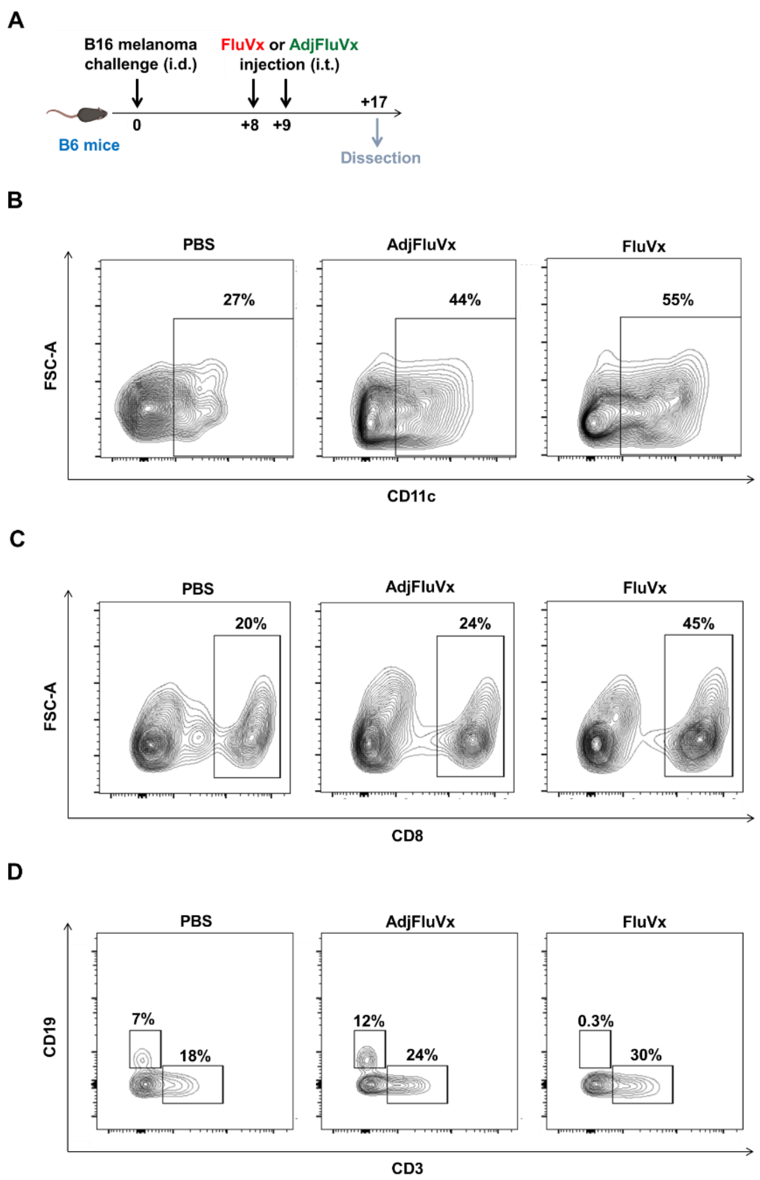

Fig. S12. Cumulative flow cytometry plots of intratumoral dendritic cells (DCs), CD8+ T cells, and ratio of B cells to T cells from mice treated with unadjuvanted or adjuvanted seasonal influenza vaccine, from experiment described in Figure 6 *A-C* and *E*. (*A*) Experimental design. Unadjuvanted seasonal influenza vaccine (FluVx): FluVx1. n = 3-5 pooled tumors/group. Data are representative of at least two independent experiments with similar results. (*B*) Cumulative flow cytometry plots of dendritic cells ($CD11c^{+}$) among intratumoral antigen presenting cells (APCs; $CD45^{+}MHC\text{-}II^{+}$) from experiment described in (*A*). (*C*) Cumulative flow cytometry plots of $CD8^{+}$ T cells ($CD8^{+}$) among intratumoral T cells ($CD45^{+}CD3^{+}$) from experiment described in (*A*). (*D*) Cumulative flow cytometry plots of B cells ($CD19^{+}$) and T cells ($CD3^{+}$) among intratumoral immune cells ($CD45^{+}$) from experiment described in (*A*). i.d., intradermal, i.t., intratumoral. PBS, phosphate-buffered saline. FSC-A, forward scatter area. AdjFluVx, adjuvanted seasonal influenza vaccine.

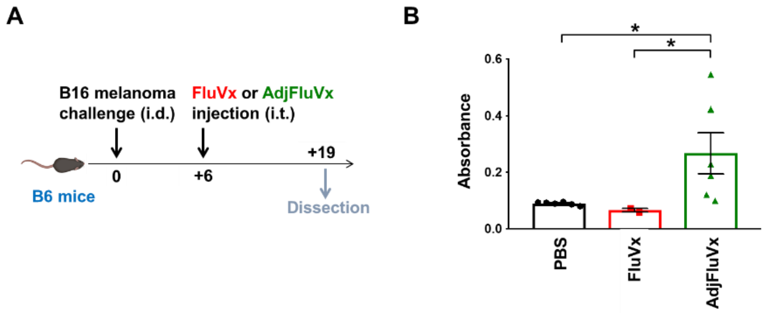

Fig. S13. Intratumoral adjuvanted seasonal influenza vaccine administration increases influenza virus-specific antibodies in the tumor. (*A*) Experimental design. Unadjuvanted seasonal influenza vaccine (FluVx): FluVx1. n = 2-6 mice / group. Data are representative of at least two independent experiments with similar results. (*B*) Bar graphs showing absorbance measured by ELISA assay from experiment described in (*A*). **P* < 0.05 [Kruskal-Wallis with Dunn's Multiple Comparisons comparing all groups to AdjFluVx (*C*)]. Error bars: mean ± s.e.m. i.d., intradermal. i.t., intratumoral. AdjFluVx, "adjuvanted" seasonal influenza vaccine.

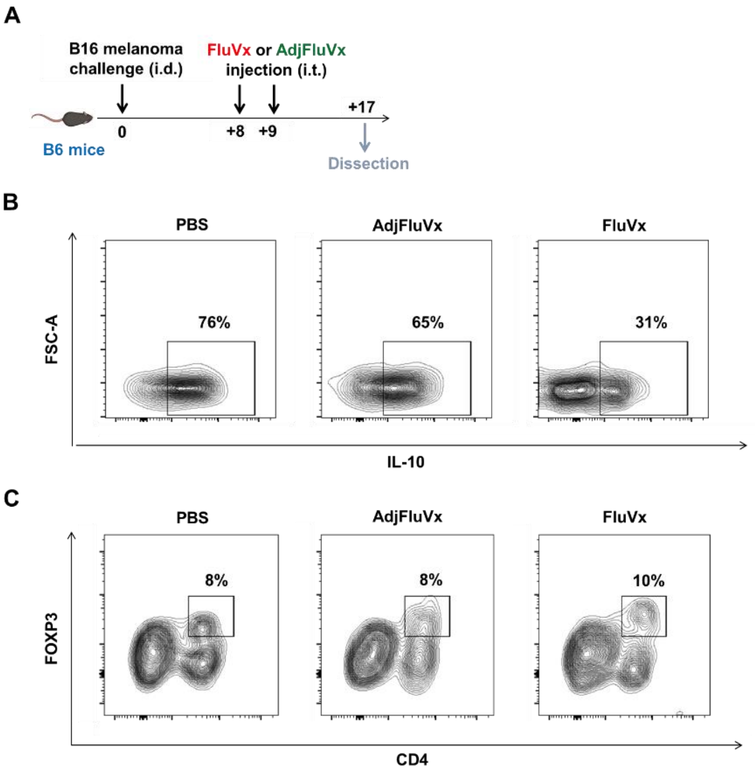

Fig. S14. Cumulative flow cytometry plots of intratumoral B regulatory cells and T regulatory cells from mice treated with unadjuvanted or "adjuvanted" seasonal influenza vaccine. (*A*) Experimental design. Unadjuvanted seasonal influenza vaccine (FluVx): FluVx1. n = 3-5 pooled tumors/group. Data are representative of at least two independent experiments with similar results. (*B*) Cumulative flow cytometry plots of regulatory B cells (Bregs; IL-10^+) among intratumoral B cells ($CD45^+CD20^+$) from experiment described in (*A*). (*C*) Cumulative flow cytometry plots of regulatory T cells (Tregs; $FOXP3^+$) among intratumoral CD4+ T cells ($CD45^+CD3^+CD4^+$) from experiment described in (*A*). i.d., intradermal. i.t., intratumoral. PBS, phosphate-buffered saline. FSC-A, forward scatter area. AdjFluVx, "adjuvanted" seasonal influenza vaccine.

Table S1. FDA-approved 2017-2018 seasonal unadjuvanted and "adjuvanted" influenza vaccines utilized in the study.

Influenza vaccine[a]	Manufacturer	Strains	HA/50 µL dose	Adjuvant	Production vehicle	Method of virus inactivation	Solvent
**FLUCELVAX® (FluVx1)**	Seqirus	Quadrivalent	6 µg (1.5 µg per strain)	No	MDCK cells	β-propiolactone and cetyltriammonium bromide	Phosphate-buffered saline
**FLUVIRIN® (FluVx2)**	Seqirus	Trivalent	4.5 µg (1.5 µg per strain)	No	Embryonated chicken eggs	β-propiolactone	Phosphate-buffered saline
**FLUARIX® (FluVx3)**	GlaxoSmithKline	Quadrivalent	6 µg (1.5 µg per strain)	No	Embryonated chicken eggs	Sodium deoxycholate and formaldehyde	Sodium chloride/sodium phosphate
**FLUBLOK® (FluVx4)**	Protein Sciences Corporation	Quadrivalent	18 µg (4.5 µg per strain)	No	Sf9 cells	Live virus never infects cells; antigens extracted using Triton-X 100	Sodium chloride/sodium phosphate
**FLUAD® (AdjFluVx)**	Seqirus	Trivalent	4.5 µg (1.5 µg per strain)	Yes (MF59®)	Embryonated chicken eggs	Formaldehyde and cetyltriammonium bromide	Oil-water emulsion (MF59®)

[a]Details regarding vaccine manufacturer, the number of influenza virus strains contained within each vaccine, concentration of hemagglutinin (HA) within each 50-µL dose, adjuvant included in the vaccine, vehicle type utilized for vaccine production, method of virus inactivation, and the vaccine solvent utilized, are provided. More information on can be obtained from each vaccine's FDA package insert, which contains full information regarding vaccine formulation and clinical data, and information regarding adjuvants in influenza vaccines can be found at: https://www.cdc.gov/vaccinesafety/concerns/adjuvants.html.

SI References

1. A. Zloza *et al.*, NKG2D signaling on CD8(+) T cells represses T-bet and rescues CD4-unhelped CD8(+) T cell memory recall but not effector responses. *Nature medicine* **18**, 422-428 (2012).
2. C. S. Carlson *et al.*, Using synthetic templates to design an unbiased multiplex PCR assay. *Nature communications* **4**, 2680 (2013).
3. H. S. Robins *et al.*, Comprehensive assessment of T-cell receptor beta-chain diversity in alphabeta T cells. *Blood* **114**, 4099-4107 (2009).
4. M. Yousfi Monod, V. Giudicelli, D. Chaume, M. P. Lefranc, IMGT/JunctionAnalysis: the first tool for the analysis of the immunoglobulin and T cell receptor complex V-J and V-D-J JUNCTIONs. *Bioinformatics* **20 Suppl 1**, i379-385 (2004).
5. R. O. Emerson *et al.*, High-throughput sequencing of T-cell receptors reveals a homogeneous repertoire of tumour-infiltrating lymphocytes in ovarian cancer. *The Journal of pathology* **231**, 433-440 (2013).
6. D. Wu *et al.*, High-throughput sequencing detects minimal residual disease in acute T lymphoblastic leukemia. *Science translational medicine* **4**, 134ra163 (2012).
7. M. C. Serradell *et al.*, Efficient oral vaccination by bioengineering virus-like particles with protozoan surface proteins. *Nature communications* **10**, 361 (2019).

DATASETS (included as separate files)

Dataset S1 (separate file). Data File for NanoString mRNA Analysis from Fig. 4C.

Dataset S2 (separate file). Data File for Adaptive ImmunoSEQ TCR Analysis from Fig. 4H.

Dataset S3 (separate file). Data File for NanoString mRNA Analysis from Fig. 5G.
